# Impact of operator expertise on transperineal free-hand mpMRI-fusion-targeted biopsies under local anaesthesia for prostate cancer diagnosis: a multicenter prospective learning curve

**DOI:** 10.1007/s00345-023-04642-2

**Published:** 2023-10-12

**Authors:** Giorgio Calleris, Alessandro Marquis, Junlong Zhuang, Mattia Beltrami, Xiaozhi Zhao, Yansheng Kan, Marco Oderda, Haifeng Huang, Riccardo Faletti, Qing Zhang, Luca Molinaro, Wei Wang, Hongqian Guo, Paolo Gontero, Giancarlo Marra

**Affiliations:** 1 Department of Urology, San Giovanni Battista Hospital, Città della Salute e della Scienza, University of Turin, C.so Bramante 88/90, 10126 Turin, Italy; 2 Department of Urology, Drum Tower Hospital, Medical School of Nanjing University, Nanjing University, Nanjing, Jiangsu People’s Republic of China; 3 Department of Radiology, San Giovanni Battista Hospital, Città della Salute e della Scienza, University of Turin, Turin, Italy; 4 Department of Pathology, San Giovanni Battista Hospital, Città della Salute e della Scienza, University of Turin, Turin, Italy

**Keywords:** Prostate cancer, Transperineal MRI-targeted fusion biopsy, Local anaesthesia, Learning curve, Expertise, Detection rate

## Abstract

**Purpose:**

Transperineal mpMRI-targeted fusion prostate biopsies (TPFBx) are recommended for prostate cancer diagnosis, but little is known about their learning curve (LC), especially when performed under local anaesthesia (LA). We investigated how operators’ and institutions’ experience might affect biopsy results.

**Methods:**

Baseline, procedure and pathology data of consecutive TPFBx under LA were prospectively collected at two academic Institutions, from Sep 2016 to May 2019. Main inclusion criterion was a positive MRI. Endpoints were biopsy duration, clinically significant prostate cancer detection rate on targeted cores (csCDR-T), complications, pain and urinary function. Data were analysed per-centre and per-operator (with ≥ 50 procedures), comparing groups of consecutive patient, and subsequently through regression and CUSUM analyses. Learning curves were plotted using an adjusted lowess smoothing function.

**Results:**

We included 1014 patients, with 27.3% csCDR-T and a median duration was 15 min (IQR 12–18). A LC for biopsy duration was detected, with the steeper phase ending after around 50 procedures, in most operators. No reproducible evidence in favour of an impact of experience on csPCa detection was found at operator’s level, whilst a possible gentle LC of limited clinical relevance emerged at Institutional level; complications, pain and IPSS variations were not related to operator experience.

**Conclusion:**

The implementation of TPFBx under LA was feasible, safe and efficient since early phases with a relatively short learning curve for procedure time.

**Supplementary Information:**

The online version contains supplementary material available at 10.1007/s00345-023-04642-2.

## Introduction

In the multiparametric prostate MRI era, prostate biopsy strategies have been revolutionised and the benefits of an MRI-based diagnostic pathway have been demonstrated with a high level of evidence [1, 2]. MRI-targeted biopsy, along with or in place of random mapping of the gland, is, therefore, becoming more and more widespread [3], and a wide variety of technical solutions for targeting and tracking a suspect MRI lesion exists [4]. Moreover, increasing antibiotic resistance has renewed the interest for the transperineal (TP) over the transrectal (TR) route, the former possibly allowing also an easier targeting of anterior lesions; the advantages of each technique have been previously described. Currently, guidelines recommend the TP route as the preferred option to reduce infectious complications [5, 6]. Several authors proved the procedure is feasible and with low morbidity under local anaesthesia [7].

Whilst some early adopters have now a relevant experience, many others will likely change the approach based on the increasing evidence in favour of the TP route. In case of TR biopsies, authors reported a relatively short learning curve [8]. When performing targeted cores through the rectum, the number of cases needed to achieve an optimal threshold does not seem to increase much [9].

However, little is known about the level of expertise needed to correctly perform a TP biopsy and, more importantly, mpMRI-targeted fusion biopsy (TPFBx). Also, the TP procedure has been historically performed mainly under general anaesthesia. Consequently, reports on the learning curve (LC) for TPFBx under LA, which are increasing their use and will likely become the standard of care in the near future, are limited.

Furthermore, available evidence usually focuses on detection rate or other prostate cancer (PCa)-related outcomes. Nonetheless, other important parameters should be considered, including procedural time, patient pain and complications, which, to our knowledge, have not been all addressed when evaluating LCs.

Our group previously published oncological and functional results of a large multicentre prospective series of TPBx under LA [5, 10]. In this work, we aim to define the LC to perform TPFBx under LA, evaluating oncological as well as procedure duration, functional outcomes and procedure-related complications.

## Patients and methods

### Study cohort and data collection

Data of patients undergoing TPFBx at San Giovanni Battista Hospital, Turin, Italy and at Drum Tower Hospital, Nanjing, China, from September 2016 to May 2019 were prospectively collected. All included patients had a positive mpMRI (PI-RADS v2 score ≥ 3) performed due to elevated PSA and/or suspicious digital rectal exam (DRE). Inclusion and exclusion criteria have already been detailed [5]. Patient characteristics, including a detailed medical history, PSA, DRE and MRI findings were recorded before biopsy.

### mpMRI imaging and pathology

All patients underwent 1.5- or 3-Tesla mpMRI study, having at least three sequences (triplanar T2-weighted, dynamic contrast-enhanced, and diffusion-weighted imaging), which were evaluated according to PI-RADS version 2.0 (and version 2.1 after 2019) score and sector map [11]**.**

### Previous experience and tutoring

No centre and/or operator had previously performed any TPFBx under LA. Centre 1 operators were used to transperineal prostate biopsy under LA and were naive to targeted approach, whilst Centre 2 operators performed transperineal targeted biopsies under general anaesthesia, as detailed in Appendix 1. Before data collection begun, all operators were trained and proctored during two TPFBx sessions under LA (around 15–20 procedures).

### Biopsy technique and histopathology

All biopsies were carried out in an outpatient setting under local anaesthesia (performing periprostatic block and subcutaneous injections, with a total of 20 mL 1% lidocaine), by a total of 30 different operators in 2 centres. Patients were given antibiotic prophylaxis and prescribed a cleansing enema before the procedure. The Esaote™ platform was employed for ultrasound images acquisition and fusion of ultrasound and mpMRI images (Esaote MyLab Machine class C, NaviSuite 5.0; Esaote, Genova, IT, for the Italian patients and Esaote Real Time Virtual Sonography, Hitachi Medical Corporation, Tokyo, Japan, for the Chinese patients). After standardised local anaesthesia, TPFBx was performed (median 2 cores per target; IQR 2–4), followed by 12 systematic cores, taken in the posterior peripheral zone, according to a pre-defined scheme. The precise steps of TP technique have been previously described [12]. All biopsies were evaluated by two dedicated senior uro-pathologist with more than 10 years’ experience in prostate pathology.

### Definition of variables

Clinically significant PCa (csPCa) was defined as Gleason score ≥ 7 in at least one biopsy core. Urinary function was assessed through the IPSS questionnaire pre-operatively and at 4 weeks after the procedure; erectile function through the IIEF-5 questionnaire pre-operatively and at 4 weeks. Peri-procedural pain was graded by interviewing the patient during the procedure at three pre-defined timepoints, using a 0–10 numeric rating scale (NRS); severe pain was defined as NRS ≥ 7. Pre-procedural anxiety was evaluated on a 11-point NRS scale, too. These assessments, their drawbacks and additional methodology details on pain assessment have been previously described [10]. Complications were categorised according to the Clavien–Dindo scale according to the EAU guidelines on reporting complications [13].

The total duration of the procedure included the following phases: (1) local anaesthesia, (2) target sampling phase, with MRI–US imaging alignment, lesion targeting and biopsy, (3) random mapping.

The clinically significant cancer detection rate on target biopsy (csCDR-T) was calculated as the fraction of positive cases on the total number of cases performed. We also considered csCDR on biopsy mapping (csCDR-M) similarly.

### Outcome definitions

Our primary outcome was to describe the existence of a LC for TPFBx, investigating “learning” variables: (i) biopsy duration (min); (ii) csCDR-T. The csCDR on standard mapping was also calculated, as a reference. Secondary outcome was to assess existence of a LC in terms of (i) biopsy-related complications; (ii) biopsy-related pain; (iii) urinary function variation. Each outcomes was assessed per-centre and per-operator. When performing per-operator analysis, we included urologist with more than 50 cases overall performed during the study period, considering the first 96 procedures, where available.

### Statistical analysis

Continuous variables were expressed as medians and interquartile range (IQR). Categorical variables were expressed as absolute numbers and/or percentages. Considering the sample size, as a preliminary analysis, we defined consecutive groups of patients (CGP) including *n* = 50 observations for per-centre analysis and *n* = 16 observations for per-operator analysis. Differences in baseline patient characteristics amongst CGPs were assessed by Kruskal–Wallis test by ranks and Pearson’s Chi-squared test. We employed Jonckheere–Terpstra test and Cochran–Armitage test to detect trends in learning continuous and categorical variables amongst CGPs, respectively.

Subsequently, univariable and multivariable logistic and linear regression models were used to identify predictors of cancer detection and procedure time, as appropriate, defining the experience as the number of procedures previously performed by a given operator or in a given centre.

To draw the LCs, a lowess smoothing function with multiple predictors (*mlowess)* was employed; this function carries out a locally weighted regression of the learning variable on operators’ experience, adjusted by other relevant predictors [14].

Finally, a cumulative sum of recursive residuals (CUSUM) test, for coefficients stability in a time-series regression, was applied to procedure time LC (continuous variable), to detect the transition from a steep phase to a slower phase/plateau of the LC. CUSUM analysis was also performed for cancer detection rate (dichotomous variable), as reported in the literature [15–17].

Statistical analyses were performed using STATA version 17.0 (StataCorp LLC, College Station, TX, USA) and SPSS, version 28.0.1 (IBM Corp., Armonk, NY, USA) and a *p* value of ≤ 0.05 was set as a significant difference. Further details on variable selection for multivariable analysis and statistical methods are available in Appendix 1.

## Results

Baseline population characteristics and main procedural outcomes are reported in Table 1. Overall, 30 operators performed 1014 TPBx under LA, 406 and 608 in Centre 1 (28 operators) and Centre 2 (2 operators), respectively. Four operators performed more than fifty TPBx under LA and were considered for per-operator analysis.

**Table 1:** Baseline patient and procedure characteristics

Baseline characteristics	Median (IQR) or *N* (%)
Age (years)	67 (62–72)
BMI (kg/m^2^)	24.7 (22.9–26.7)
Positive family history	74 (7.3)
Charlson comorbidity score	
≤ 2	732 (72.2)
3–4	255 (25.1)
≥ 5	26 (2.6)
ASA	
1	306 (30.2)
2	673 (66.4)
≥ 3	35 (3.5)
ECOG	
0	318 (31.4)
1	361 (35.6)
2	327 (32.2)
≥ 3	8 (0.8)
PSA (mg/dL)	7 (5.1–10.3)
Prostate volume (cc)	45.3 (31.4–62.5)
PSA density (mg/dL/cc)	0.16 (0.24–0.37)
Positive DRE	237 (23.4)
Number of MRI targets	
1	672 (66.3)
2	274 (27)
3	68 (6.7)
PI-RADS score (main target)	
3	366 (36.7)
4	494 (49.6)
5	136 (13.7)
Location (main target)	
Anterior	412 (40.6)
Posterior	569 (56.1)
Both	33 (3.3)
Diameter (main target, mm)	10 (7–13)
csPCa on target biopsy	277 (27.3)
csPCa on biopsy	359 (35.4)
ISUP GG on target biopsy	
neg	639 (63)
1	98 (9.7)
2	137 (13.5)
3	96 (9.5)
≥ 4	44 (4.3)
Procedure time (min)	15 (12–18)
Anxiety (NRS 0–10)	3 (2–4)
Maximum pain (NRS 0–10)	4 (3–6)
Complications, *N*	73 (7.2%)
Severe complications, *N*	0 (0%)
IPSS score difference	0 (0–0)

*IQR* interquartile range, *N* number of patients, *BMI* body mass index, *PCa* prostate cancer, *ASA* American Society of Anesthesiology, *ECOG PS* Eastern Cooperative Oncology Group Performance Status scale, *DRE* digital rectal exam, *MRI* magnetic resonance imaging, *csPCa* clinically significant PCa, *NRS* numerical rating scale, *IPSS difference* International Prostatic Symptoms Score difference (30 days after the procedure and at baseline), *ISUP GG* International Society of Urological Pathology grade group

csCDR-T was 27.3%, overall procedural time 15 min (IQR 12–18), median maximum procedure-related pain was 4 (IQR 3–6) and complications were experienced by 7.2% of patients. No cases of severe complications or urosepsis were recorded.

### Procedure duration

A significant trend toward a shorter procedure duration is visible across consecutive patients groups for Centre 2 and for Operators 1, 2, 3, whilst Operator 4 shows a borderline *p* value (Supplementary Tables 1, 4, 5).

Univariable regression analysis results are available in Supplementary Table 2. In multivariable analysis, operators’ experience is a predictors of procedure duration for Operators 1, 2 and 4 (Table 2).

**Table 2:** Multivariable linear regression model for biopsy total duration on target biopsy, per-centre and per-operator (Op)

Biopsy time multivariable regression	Centre 1	Centre 2	Operator 1	Operator 2	Operator 3	Operator 4
Centre experience						
Coef	− 0.002	− 0.006				
*p*	*0.36*	**0.001**				
Operator experience						
Coef			− 0.15	− 0.044	− 0.01	− 0.049
*p*			**0.001**	**0.016**	*0.109*	**0.001**
Age						
Coef	− 0.023	0.035	− 0.307	0.1	0.026	0.033
*p*	*0.576*	**0.011**	**0.006**	*0.169*	*0.274*	*0.473*
Number of MRI targets						
Coef	3.103	2.074	6.806	1.083	2.422	2.452
*p*	**0.001**	**0.001**	**0.001**	*0.369*	**0.001**	**0.001**
Pain NRS (0–10)						
Coef	0.299	− 0.051	0.328	0.339	− 0.208	− 0.076
*p*	**0.011**	*0.339*	*0.272*	*0.086*	*0.053*	*0.652*
PI-RADS 4 lesion^a^						
Coef	0.797	0.404	− 2.29	1.66	− 0.197	0.244
*p*	*0.32*	*0.065*	*0.253*	*0.209*	*0.649*	*0.716*
PI-RADS 5 lesion^a^						
Coef	− 0.079	0.668	− 5.24	1.471	0.9	− 0.375
*p*	*0.937*	**0.042**	**0.028**	*0.367*	*0.143*	*0.697*
Prostate volume [cc]						
Coef	0.004	− 0.012	− 0.03	− 0.005	− 0.006	− 0.014
*p*	*0.723*	**0.001**	*0.289*	*0.741*	*0.204*	*0.411*

The 95% confidence intervals for this table are shown in Supplementary Table 6A

Significant *p* values (below 0.05) are in bold

*MRI* magnetic resonance imaging, *NRS* numerical rating scale, *PI-RADS* Prostate Index Reporting and Data System score, *Coef.* adjusted beta coefficient, *95% CI* 95% confidence interval

^a^Reference is PI-RADS 3 lesion

**Fig. 1:**
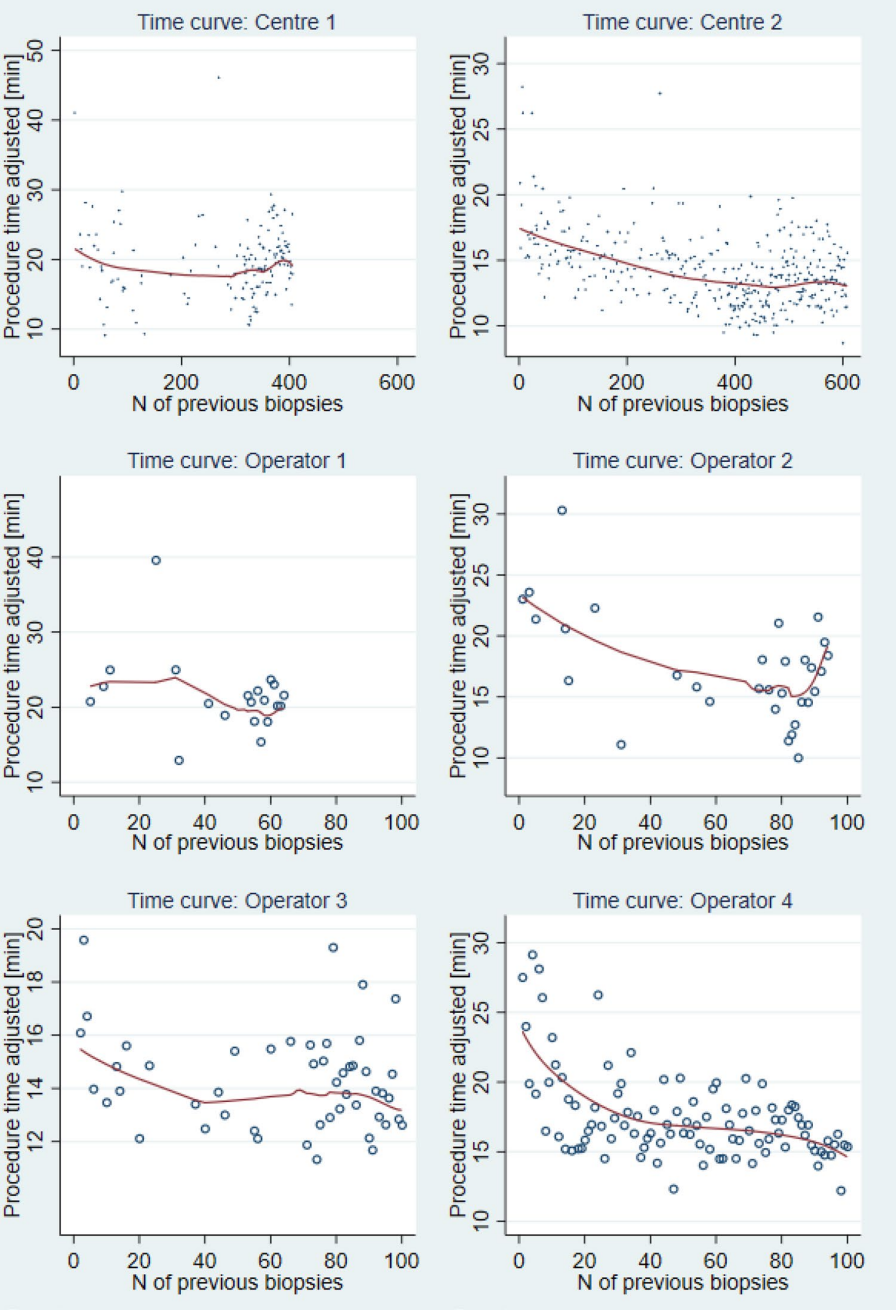
Biopsy duration learning curve (*mlowess* function), adjusted for patient pain, number of targets, total biopsy cores, PI-RADS score. “N of previous biopsies” is the number of TPFBx under LA previously performed in a centre or by an operator (at study start, zero TPFBx under LA had been performed in both centres and by all operators)

Figure 1 depicts the LCs for biopsy duration adjusted for patient pain, number of targets, total biopsy cores, PI-RADS score. In accordance with multivariable analysis, a trend is visualised for all operators, with a steeper initial phase.

In CUSUM per-operator analysis (Supplementary Fig. 1), a significant change in regression coefficients is detected after 50 procedures for Operator 2 and 4, which is to be interpreted as a transition from a steep learning phase to a slower learning or plateau phase. Operator 3 approaches statistical significance around 50 procedures, whilst for Operator 1, no change from linear trend is detected.

### csCDR-T

Analysing consecutive patients groups, a significant trend for improved csCDR-T was observed only for Centre 2 (Supplementary Table 1).

Univariable regression analysis results are available in Supplementary Table 3. In multivariable analysis, experience was significantly associated with csCDR-T only for Centre 2 (Table 3). Predictors were not reproducible for all centres and all operators.

**Table 3:** Multivariable logistic regression model for csPCa detection rate on target biopsy (csCDR-T), per-centre and per-operator

csCDR-T-multivariable regression	Centre 1	Centre 2	Operator 1	Operator 2	Operator 3	Operator 4^b^
Age						
OR	1.082	1.081	1.045	1.176	1.004	1.332
*p*	**0.001**	**0.001**	*0.48*	**0.003**	*0.95*	**0.042**
Centre experience						
OR	1.002	1.004	-	-	-	-
*p*	*0.088*	**0.001**				
Operator experience						
OR	-	-	0.999	1.023	0.984	1.037
*p*			*0.984*	*0.075*	*0.427*	*0.094*
PSA density [ng/mL/cc]						
OR	222.952	43.973	0.722	3.120.352	46.474	2.840.006
*p*	**0.002**	**0.001**	*0.926*	**0.043**	*0.237*	*0.057*
Prostate volume [cc]						
OR	0.979	0.98	0.946	0.99	0.97	0.955
*p*	**0.001**	**0.024**	**0.019**	*0.407*	*0.402*	*0.266*
Positive DRE						
OR	2.376	2.954	5.297	5.643	1.961	26.384
*p*	**0.002**	**0.001**	**0.039**	**0.013**	*0.555*	**0.027**
PI-RADS 4 lesion^a^						
OR	3.861	5.224	1.73	5.383	3.709	3.997
*P*	**0.001**	**0.001**	*0.633*	*0.05*	*0.314*	*0.451*
PI-RADS 5 lesion^a^						
OR	3.314	8.444	71.576	3.931	2.084	237.558
*p*	**0.025**	**0.001**	**0.037**	*0.213*	*0.671*	**0.014**
Target diameter [mm]						
OR	1.157	1.009	0.837	1.245	1.087	0.887
*p*	**0.001**	*0.752*	*0.161*	**0.01**	*0.353*	*0.294*
Positive family history						
OR	0.812	3.822	0.211	0.825	-	61.396
*p*	*0.587*	**0.023**	*0.224*	*0.809*		**0.008**

The 95% confidence intervals for this table are shown in Supplementary Table 6B

Significant *p* values (below 0.05) are in bold

*DRE* digital rectal examination, *PI-RADS* Prostate Index Reporting and Data System score, *OR* odds ratio

^a^Reference is PI-RADS 3 lesion

^b^Firth procedure for logistic regression used for Operator 4

**Fig. 2:**
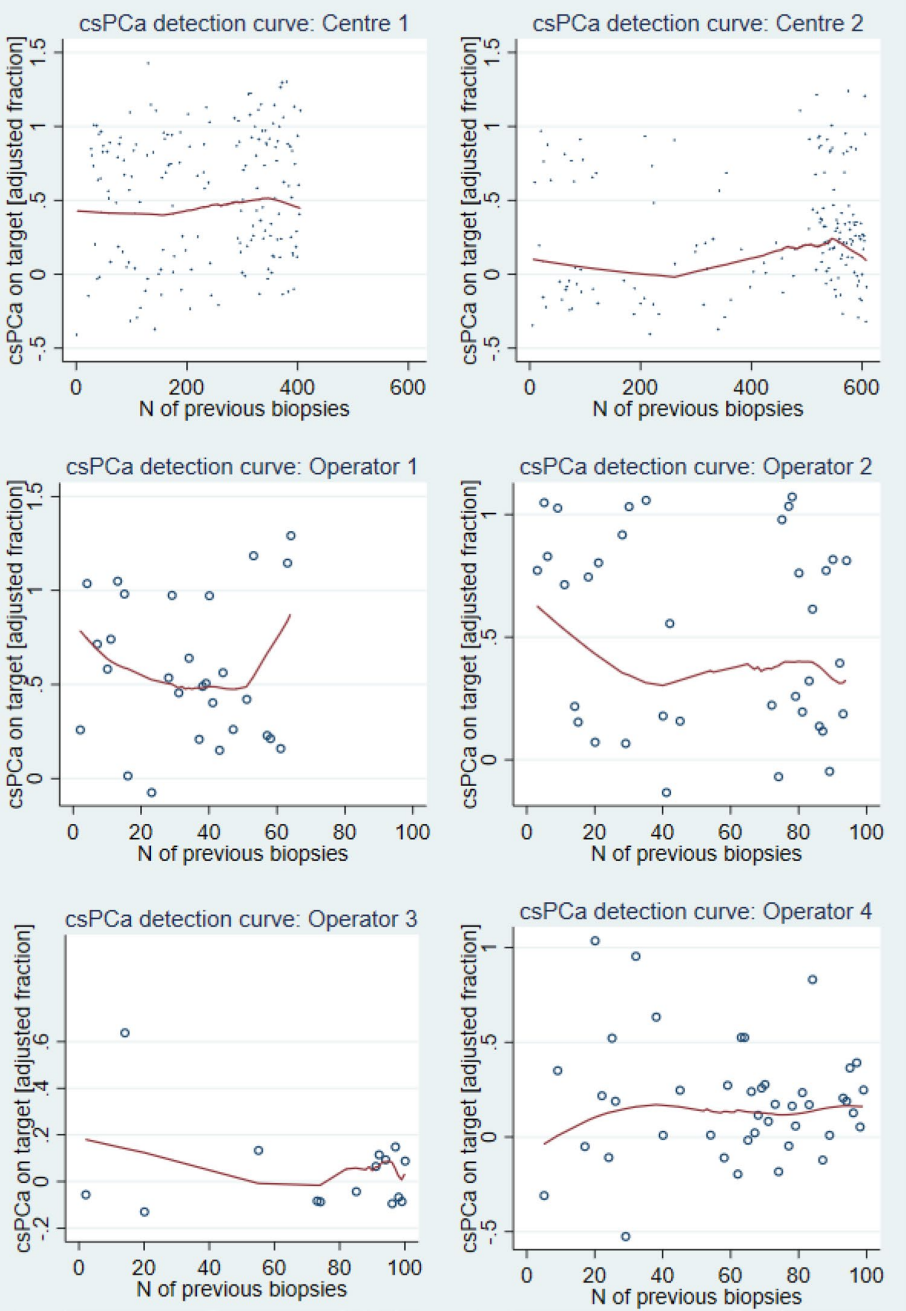
Learning curves for csPCa detection rate on target biopsy (*mlowess* function), adjusted for age, PSA density, PI-RADS score and digital rectal examination (DRE). “N of previous biopsies” is the number of TPFBx under LA previously performed in a centre or by an operator (at study start, zero TPFBx under LA had been performed in both centres and by all operators)

Figure 2 depicts the LCs for csCDR-T, adjusted for age, PSA density, PI-RADS score and DRE. A visual, non-significant trend can be seen for Operator 4, whilst the slope in the per-centre curves appears very slight.

In CUSUM per-operator analysis, a declining non-statistically significant trend is visualised after around 80 procedures (Supplementary Fig. 2).

### Complications, pain and urinary function

Complications overall incidence was 7.2% (all were Clavien–Dindo grade ≤ 2). Amongst these, urine retention, non-completed procedure, vasovagal reaction and perineal bleeding were the most frequent, representing 23.0%, 17.6%, 17.6% and 16.2% of the total, respectively.

No significant trend for procedure complications amongst consecutive patients groups was detected, neither in per-centre nor in per-operator analysis (all Cochran–Armitage *p* ≥ 0.15, data not shown).

Median overall peri-procedural pain was 4 (IQR 3–6). No significant trend for peri-procedural pain amongst consecutive patients groups was detected, neither in per-centre nor in per-operator analysis, except for one single operator (Jonckheere–Terpstra *p* = 0.03 for Operator 2; all other *p* ≥ 15%); when peri-procedural pain was adjusted for pre-procedural anxiety, regression analysis did support the existence of a LC for this operator (*p* = 0.10, data not shown).

Overall, median IPSS score difference 1 month after and before the biopsy was 0 (IQR 0–0). No evidence in favour of a LC was found at consecutive patient group analysis, neither per-centre nor per-operator (all Jonckheere–Terpstra *p* > 0.13, data not shown).

## Discussion

In this work, we analysed the impact of centre’s and operator’s experience on multiple endpoints of TPFBx under local anaesthesia. To our knowledge, we are amongst the first who considered both procedural (biopsy duration) and efficacy outcomes (complications, pain and IPSS change) other than cancer detection, rendering a more comprehensive representation of this procedure than currently reported in the literature for TPFBx under LA and for similar procedures, too [18–22]. Several findings are indeed of interest.

First, as logically expected, there is a progressive reduction in total biopsy time. This finding is consistent and reproducible, i.e. is present in three out of four operators and in Centre 2, in multivariable analysis. In Centre 1, the existence of a LC at institutional level is probably masked by the higher number of operators. The graphical analysis of the curves (Fig. 1) and the CUSUM analysis (Supplementary Fig. 1) suggest the end of the steeper learning phase after about 50 procedures. Interestingly, this threshold is statistically significant for two operators (belonging to different centres, with a different baseline experience about targeting and anaesthesia approaches) but is only “visually” detectable for the other two; possible explanations involve insufficient statistical power and/or smaller learning effect due to individual characteristics. This slightly contrasts with a threshold of 18 but is in line with the one of 42 biopsies previously reported for in-bore biopsies under general anaesthesia and transrectal fusion biopsies under sedo-analgesia, respectively [19, 21]. Moreover, we did not notice different results patterns based on baseline experience with targeted biopsy under general anaesthesia.

Second, the existence of a LC for cancer detection is elusive and, if present, likely of limited clinical impact. In multivariable analysis, experience did not reach statistical significance as a predictor of csCDR-T at a per-operator level; a visual trend might be detected in Operator 4 learning curve (Fig. 2). CUSUM analysis does not reach significance at operator’s level, speaking against the existence of a LC for CDR in this setting; inspecting CUSUM charts, a 80- to 90-procedure threshold may be hypothesised as a transition point to a plateau phase, especially for Operators 2 and 4. Conversely, at Institutional level, centre experience is a predictor of csCDR-T in multivariable analysis for Centre 2, even though the difference in csCDR-T amongst last and first CPGs appears of limited clinical relevance (+ 1% in Centre 1 and + 4% in Centre 2). This might suggest the existence of a hypothetical gentle and longer LC, influenced by several factors amongst which a fluctuation in disease prevalence, the operator’s individual characteristics and, above all, a parallel radiological LC (on both radiologist’s and urologist’s sides) are to be considered [19, 23, 24]. Lesion location (anterior or posterior) did not impact detection rates: this finding suggests a good sampling of the anterior targets by the TP technique.

Third and reassuringly, our data provide no evidence in favour of an increased risk of complications, procedure-related pain or decreased urinary function (measured by IPSS) in early phases, for centres or operators adopting TPFBx under LA. As previously reported, no serious complications were reported and the tolerability of the procedure was good, with only 13 cases (1.3%) interrupted and rescheduled under general anaesthesia [5, 10]. We acknowledge that a longer biopsy time has been associated with increased pain [10]; although we showed that increasing operator’s experience can reduce biopsy duration, we could not prove any association with patient pain. On the one hand, we must consider that operators underwent a two-session proctoring and used a standardised anaesthesia technique [12]. On the other hand, other factors (e.g. patient anxiety, number of targets, prostate volume, lesion location, technical issues) might play a more important role in this regard whilst operator’s experience likely has a non-clinically meaningful impact on patient pain, with appropriate previous experience and/or proctoring on the LA technique.

In general, to evaluate a learning process in surgical procedures, multiple factors have to be taken into account (e.g. technology characteristics, centres, population and operator characteristics) [25]. Moreover, in the case of prostate biopsy, the main efficacy outcome of the procedure (i.e. cancer detection) cannot be tested against a definitive gold standard and is, therefore, deeply influenced by the stochastic disease prevalence and distribution in the included sample. These issues were particularly evident in our population, considering also the differences between the two institutions which, however, might help the generalisability of our results. The literature reports heterogeneous and sometimes contradictory results about the learning curve for cancer detection in similar biopsy procedures. For instance, Halstuch and coll. have reported a single-surgeon prospective series, describing a LC being steeper for the first 100 procedures circa and similar both for transrectal and transperineal approaches, the latter performed under general anaesthesia [20]. Stabile and coll. also have shown an impact of operators’ expertise on cancer detection rate, but possibly more pronounced for transrectal than for transperineal fusion biopsies with a steep phase of about 60 cases [18]. Hsieh et al. reported that TPFBx under general anaesthesia learning curve shows significant improvement over a 4-year period [26]. Meng and coll. reported a nearly doubled detection rate after a 4-year period using TR fusion biopsy under LA [27]. Mager et al. retrospectively examined the TR fusion biopsy under sedo-analgesia of a novel and expert operators, reporting an improved detection and decreased time after 42 procedures, whilst Cata and coll. identified a detection plateau after 52 cases under LA [19, 28]. Rosenzweig and coll. analysed and found no evidence for a cancer detection LC considering in-bore MRI-guided biopsy under general anaesthesia. Kasabwala et al., although reporting an experience-related improvement of needle trajectory and pathological quality for TR fusion biopsies, did not observe any change in csPCa detection over time [22]. Westhoff and coll. identified a minimum experience threshold of 8 procedure for transrectal approach, with similar target biopsy results for experienced senior physicians and residents [9].

To address these issues, we have investigated the LCs through several independent statistical methods on prospectively acquired data: CPGs fractions and medians trends, logistic/linear regression and CUSUM analyses; this overcomes the limitation imposed by arbitrarily defined group segmentation of other similar reports and enhances the robustness of our findings [19, 28]. Our work is not devoid of limitations: no central MRI review was performed and heterogeneity in imaging was not assessed; the number of operators, their previous experience and the disease prevalence were different amongst included centres; the length of the biopsy series considered for each operator is limited, and therefore our results might reflect mainly the early phase of the learning curve; a single fusion biopsy platform (Esaote) was employed; no analysis on biopsy needle trajectory, percentage of tumour on positive cores, ratio of upgrading at final pathology was performed [22, 26].

From the clinical perspective, we found that the existence of a relatively short LC for procedure duration is sufficiently reproducible in per-operator analyses, whilst the LC for cancer detection is more elusive, being detected only in one Institution, and is probably of a limited magnitude; moreover, no evidence for a LC was found when considering complications and functional outcomes. Our results seem not radically different if compared to those referred to similar biopsy procedures under general anaesthesia and/or with a transrectal approach. Therefore, the implementation of TPFBx under LA, after a short training/supervision period, should be encouraged as a safe, tolerable and efficient technique since early phases. Our findings advocate against the myth of difficulties in implementing TP biopsies under LA [29].

From a research perspective, future studies willing to prospectively assess the LC for TPFBx under LA might focus on totally biopsy-naïve operators exposed to a standardised training period and ideally employ MRI interpreted by a senior radiologists, to reduce bias.

## Conclusion

In operators already performing TP biopsy under LA but naïve to the fusion approach, or already performing TPFBx but under general anaesthesia, the implementation of TPFBx under LA was feasible, safe and efficient since early phases. A LC for biopsy duration was detected, with the steeper phase ending after around 50 procedures, in most cases. However, experience did not impact csPCa detection at an operator’s level, whilst a possible longer and gentle LC of limited clinical relevance emerged at Institutional level; complications, pain and IPSS variations were not related to operator experience.

### Supplementary Information

Below is the link to the electronic supplementary material.

Supplementary file1 (DOCX 35 KB)

Supplementary file2 (DOCX 60 KB)

Supplementary file3 (DOCX 126 KB)

Supplementary file4 (DOCX 20 KB)

Supplementary file5 (DOCX 29 KB)

Supplementary file6 (DOCX 32 KB)

Supplementary file7 (DOCX 39 KB)

Supplementary file8 (DOCX 39 KB)

Supplementary file9 (DOCX 25 KB)

## Data Availability

The data presented in this study are available on motivated request from the corresponding author.
