# Supplementary material for: Impact of operator expertise on transperineal free-hand mpMRI-fusion-targeted biopsies under local anaesthesia for prostate cancer diagnosis: a multicenter prospective learning curve
Source: World J Urol. 2023 Oct 12;41(12):3867–76. doi: 10.1007/s00345-023-04642-2 (PMC10693515; doi:10.1007/s00345-023-04642-2)
Supplement: Supplementary file 1 — Supplementary file1 (DOCX 35 KB) [file 345_2023_4642_MOESM1_ESM.docx]

**Appendix 1. Additional Materials and Methods**

**1.1 Baseline biopsy experience and proctoring detail of included operators**

The following table reports the included operators’ experience with different biopsy techniques, before the data collection begun.

| Operator | Age | Centre | Baseline experience with TP fusion Bx under LA (subject of current study) | Proctored sessions of TP fusion Bx under LA (circa 7-10 procedures each) | Baseline experience with TP fusion Bx under GA | Baseline experience with TP systematic Bx under LA |
| --- | --- | --- | --- | --- | --- | --- |
| Operator 1 | Late-20s | Centre 1 | None | 2 | None | Beginner |
| Operator 2 | Late-40s | Centre 1 | None | 2 | None | Expert |
| Operator 3 | Mid-40s | Centre 2 | None | 2 | Expert | None |
| Operator 4 | Mid-40s | Centre 2 | None | 2 | Expert | None |
| Legend: TP, transperineal; Bx, prostate biopsy; LA, local anaesthesia; GA, general anaesthesia; Beginner: 20 -100 procedures; Intermediate: 101 - 500 procedures; Expert: > 500 procedures. | | | | | | |

In Centre 1, one operator was supervised during two biopsy sessions (before data collection) by a company specialist and subsequently instructed other operators. An operator of Centre 1 (G.M) proctored Centre 2 (one biopsy session under local anaesthesia and supervision of a second session). At the study end, Operators 1, 2, 3 and 4 had performed 64, 96, >100 and >100 TP fusion biopsies under LA, respectively.

**1.2 Additional statistical methods**

**1.2.1 Use of Jonckheere-Terpstra (JT) test**. The JT test is a well-established non-parametric test for differences in a quantitative variable among independent samples across ordered categories (i.e. for trends across ordered categories, when ordering of the groups is possible *a priori*). In our case, the alternative hypothesis is that biopsy time does not increase across consecutive groups of patients (CGP) and actually it decreases, at least in between two CGPs. One advantage is that JT test does not a priori hypothesise a particular shape of the relationship (Lunneborg, 2014).

**1.2.2 Selection of variables for multivariable regression models.** Variable selection for multivariable regression models was helped using a supervised forward stepwise method, at a per-centre level. F-test probability (for linear models) and likelihood ratio (for logistical models) thresholds to insert and to remove a variable were 0.05 and 0.10, respectively. We compared the included and the excluded variables with those suggested by biological plausibility and theorical knowledge, and no major changes were deemed necessary. To ensure the coherence throughout the whole work, we used the same predictors identified as described above in all the per-centre and all per-operator models.

**1.2.3 Firth’s logistic regression.** Firth’s logistic regression was used for dealing with quasi-separation issues at a per-operator level, when logistic regression was not applicable (Puhr et al., 2017). Its use is clearly identified in the legends.

**1.3 References**

Lunneborg, C. E. (2014). Jonckheere–Terpstra Test. Wiley StatsRef: Statistics Reference Online. https://doi.org/10.1002/9781118445112.STAT06337

Puhr, R., Heinze, G., Nold, M., Lusa, L., & Geroldinger, A. (2017). Firth’s logistic regression with rare events: accurate effect estimates and predictions? Statistics in Medicine, 36(14), 2302–2317. https://doi.org/10.1002/SIM.7273
