# Supplementary material for: Impact of operator expertise on transperineal free-hand mpMRI-fusion-targeted biopsies under local anaesthesia for prostate cancer diagnosis: a multicenter prospective learning curve
Source: World J Urol. 2023 Oct 12;41(12):3867–76. doi: 10.1007/s00345-023-04642-2 (PMC10693515; doi:10.1007/s00345-023-04642-2)

**Supplementary Figure 1.** CUSUM analysis of linear regression coefficient stability for biopsy time per-operator. Operator 2 and 4 diverge from linear trend after 50 procedures.


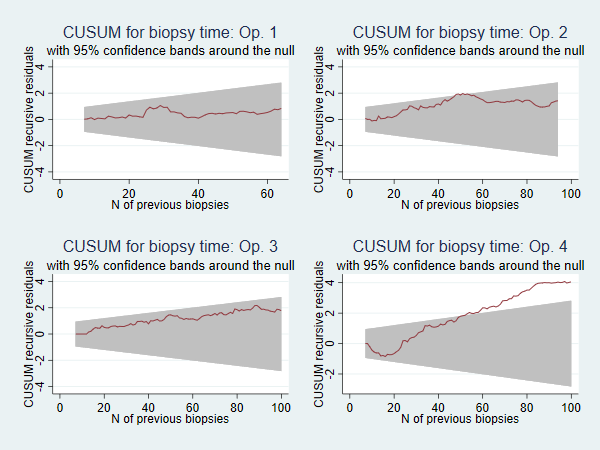

Supplement: Supplementary file 2 — Supplementary file2 (DOCX 60 KB) [file 345_2023_4642_MOESM2_ESM.docx]
