# Supplementary material for: Impact of operator expertise on transperineal free-hand mpMRI-fusion-targeted biopsies under local anaesthesia for prostate cancer diagnosis: a multicenter prospective learning curve
Source: World J Urol. 2023 Oct 12;41(12):3867–76. doi: 10.1007/s00345-023-04642-2 (PMC10693515; doi:10.1007/s00345-023-04642-2)

**Supplementary Figure 2.** CUSUM per-operator analysis of clinically significant prostate cancer (csPCa) detection rate on target. A declining trend is visualized after around 80 procedures for operators 2 and 4. All *p* are non-significant (respectively, 0.79, 0.23, 0.74, 0.79).


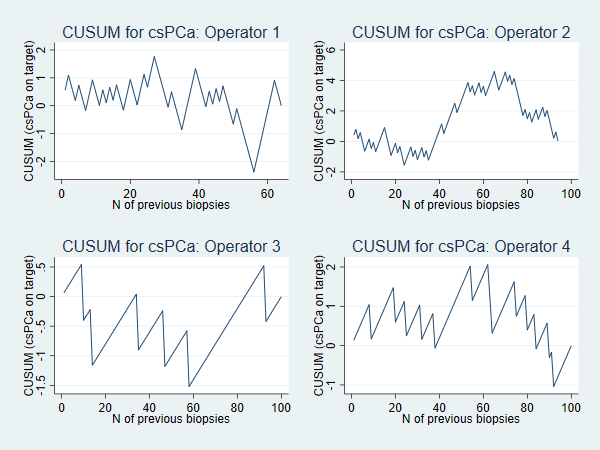

Supplement: Supplementary file 3 — Supplementary file3 (DOCX 126 KB) [file 345_2023_4642_MOESM3_ESM.docx]
