# Supplementary material for: Impact of operator expertise on transperineal free-hand mpMRI-fusion-targeted biopsies under local anaesthesia for prostate cancer diagnosis: a multicenter prospective learning curve
Source: World J Urol. 2023 Oct 12;41(12):3867–76. doi: 10.1007/s00345-023-04642-2 (PMC10693515; doi:10.1007/s00345-023-04642-2)
Supplement: Supplementary file 4 — Supplementary file4 (DOCX 20 KB) [file 345_2023_4642_MOESM4_ESM.docx]

**Supplementary Table 1.** Synoptic view of differences among consecutive patients groups in learning variables and patient variables, per centre and per operator. Significant p values are reported in green. Learning curve variables are tested for a trend across ordered groups (Cochrane-Armitage test or Jonckheere-Terpstra test, *p trend*). Patient variables are tested for differences among groups (Pearson Chi-square or Kruskal-Wallis test, *p diff*). csPCa = clinically significant prostate cancer; BMI = bosy mass index; DRE = digital rectal examination; PIRADS = Prostate Index Reporting and Data System; A = anterior; P = Posterior; NRS = numerical rating scale.

|  |  | Centre 1 | Centre 2 | Operator 1 | Operator 2 | Operator 3 | Operator 4 |
| --- | --- | --- | --- | --- | --- | --- | --- |
| N of consecutive patient groups | | 8 | 12 | 4 | 6 | 6 | 6 |
| N of patients in each group | | 50 | 50 | 16 | 16 | 16 | 16 |
| Learning variable | | **P values for trend across groups** | | | | | |
| csPCa on target biopsy | | 0,201 | 0,001 | 0,736 | 0,35 | 0,459 | 0,431 |
| csPCa on biopsy mapping | | 0,062 | 0,002 | 0,264 | 0,456 | 0,423 | 0,611 |
| Procedure time | min | 0,717 | 0,001 | 0,002 | 0,001 | 0,004 | 0,063 |
| Patient variable | | **P values for differences among groups** | | | | | |
| Age | years | 0,516 | 0,794 | 0,192 | 0,311 | 0,686 | 0,667 |
| BMI | kg/m2 | 0,075 | 0,189 | 0,084 | 0,747 | 0,878 | 0,179 |
| Family history | + | 0,636 | 0,829 | 0,249 | 0,308 | 0,99 | 0,39 |
| DRE | + | 0,132 | 0,001 | 0,415 | 0,361 | 0,76 | 0,086 |
| PSA | ng/ml | 0,269 | 0,083 | 0,984 | 0,044 | 0,975 | 0,879 |
| Prostate volume | cc | 0,299 | 0,066 | 0,102 | 0,358 | 853 | 0,397 |
| PSA density | ng/ml/cc | 0,09 | 0,011 | 0,92 | 0,132 | 0,73 | 0,594 |
| PIRADS target 1 | 3 / 4 / 5 | 0,76 | 0,225 | 0,287 | 0,19 | 0,136 | 0,389 |
| Target 1 diameter | mm (max) | 0,296 | 0,03 | 0,886 | 0,836 | 0,453 | 0,568 |
| Target location | A / P / A+P | 0,063 | 0,018 | 0,322 | 0,348 | 0,475 | 0,413 |
| Pain NRS | 0-10 | 0,003 | 0,057 | 0,834 | 0,014 | 0,904 | 0,862 |
| Anxiety NRS | 0-10 | 0,028 | 0,42 | 0,59 | 0,042 | 0,038 | 0,428 |
