# Supplementary material for: Impact of operator expertise on transperineal free-hand mpMRI-fusion-targeted biopsies under local anaesthesia for prostate cancer diagnosis: a multicenter prospective learning curve
Source: World J Urol. 2023 Oct 12;41(12):3867–76. doi: 10.1007/s00345-023-04642-2 (PMC10693515; doi:10.1007/s00345-023-04642-2)
Supplement: Supplementary file 5 — Supplementary file5 (DOCX 29 KB) [file 345_2023_4642_MOESM5_ESM.docx]

**Supplementary Table 2.** Univariable linear regression analysis for total procedure time, per centre and per operator (Op). Significant coefficients are highlighted in green (*** p<0.01, ** p<0.05, * p<0.1). NRS = numerical rating scale; BMI = body mass index; DRE = digital rectal examination; PIRADS = Prostate Index Reporting and Data System score; ASA = American Society of Anesthesiology score; ECOG PS = Eastern Cooperative Oncology Group Performance Status;§= reference is PIRADS 3 lesion; §§ = reference is anterior lesion; §§§ = reference is ASA score 1; §§§§ = reference is ECOG PS 0.

| Univariable regression |  | Centre1 | Centre2 | Op1 | Op2 | Op3 | Op4 |
| --- | --- | --- | --- | --- | --- | --- | --- |
| Age | Coeff | -0.0201 | 0.0375** | -0.225* | 0.0672 | -0.00133 | 0.0592 |
|  | 95%CI | (-0.0954 - 0.0552) | (0.00414 - 0.0708) | (-0.474 - 0.0250) | (-0.0686 - 0.203) | (-0.0585 - 0.0559) | (-0.0463 - 0.165) |
|  | P | 0.600 | 0.0276 | 0.0769 | 0.328 | 0.963 | 0.268 |
| Centre experience | Coeff | -0.00303 | -0.00793*** | -0.0358** | -0.0159*** | -0.0184*** | -0.0392*** |
|  | 95%CI | (-0.00768 - 0.00162) | (-0.00921 - -0.00666) | (-0.0653 - -0.00618) | (-0.0250 - -0.00678) | (-0.0312 - -0.00567) | (-0.0615 - -0.0170) |
|  | P | 0.200 | 0 | 0.0186 | 0.000812 | 0.00512 | 0.000710 |
| Operator experience | Coeff | -0.0580*** | -0.0101*** | -0.119*** | -0.0587*** | -0.0214*** | -0.0419*** |
|  | 95%CI | (-0.0811 - -0.0349) | (-0.0121 - -0.00818) | (-0.203 - -0.0342) | (-0.0919 - -0.0255) | (-0.0372 - -0.00565) | (-0.0654 - -0.0184) |
|  | P | 1.22e-06 | 0 | 0.00668 | 0.000687 | 0.00828 | 0.000611 |
| Pain NRS (0-10) | Coeff | 0.362*** | -0.119* | 0.0380 | 0.436** | -0.281** | -0.108 |
|  | 95%CI | (0.142 - 0.582) | (-0.255 - 0.0166) | (-0.670 - 0.746) | (0.0575 - 0.815) | (-0.557 - -0.00468) | (-0.497 - 0.282) |
|  | P | 0.00130 | 0.0852 | 0.915 | 0.0245 | 0.0463 | 0.584 |
| Positive family history | Coeff | 0.496 | 0.755 | 4.172 | -0.688 | - | -0.958 |
|  | 95%CI | (-1.138 - 2.130) | (-0.614 - 2.124) | (-1.801 - 10.14) | (-3.303 - 1.928) |  | (-4.257 - 2.341) |
|  | P | 0.551 | 0.279 | 0.168 | 0.603 | - | 0.566 |
| Consecutive patient group | Coeff | -0.155 | -0.395*** | -1.639** | -0.809*** | -0.854*** | -1.560*** |
|  | 95%CI | (-0.388 - 0.0780) | (-0.459 - -0.331) | (-3.110 - -0.169) | (-1.267 - -0.352) | (-1.416 - -0.293) | (-2.678 - -0.442) |
|  | P | 0.192 | 0 | 0.0295 | 0.000689 | 0.00322 | 0.00672 |
| Charlson score | Coeff | 0.0931 | -0.157 | -0.775 | 0.163 | -0.318 | -0.170 |
|  | 95%CI | (-0.414 - 0.600) | (-0.466 - 0.153) | (-2.424 - 0.873) | (-0.734 - 1.060) | (-0.934 - 0.298) | (-1.160 - 0.820) |
|  | P | 0.718 | 0.320 | 0.351 | 0.719 | 0.308 | 0.734 |
| BMI [kg/m2] | Coeff | 0.0297 | -0.0495 | 0.229 | -0.185 | -0.0277 | 0.138 |
|  | 95%CI | (-0.143 - 0.202) | (-0.140 - 0.0410) | (-0.270 - 0.729) | (-0.471 - 0.101) | (-0.221 - 0.165) | (-0.104 - 0.379) |
|  | P | 0.735 | 0.283 | 0.362 | 0.203 | 0.776 | 0.260 |
| PSA [ng/ml] | Coeff | -0.0116 | -0.0466 | -0.0104 | 0.0261 | 0.107* | -0.0861 |
|  | 95%CI | (-0.165 - 0.142) | (-0.105 - 0.0116) | (-0.405 - 0.384) | (-0.269 - 0.321) | (-0.00743 - 0.221) | (-0.295 - 0.123) |
|  | P | 0.882 | 0.116 | 0.958 | 0.861 | 0.0665 | 0.415 |
| Prostate volume [cc] | Coeff | 0.00273 | -0.0167*** | 0.0237 | -0.00754 | -0.0102* | 0.000469 |
|  | 95%CI | (-0.0195 - 0.0250) | (-0.0255 - -0.00787) | (-0.0435 - 0.0910) | (-0.0376 - 0.0225) | (-0.0220 - 0.00162) | (-0.0360 - 0.0369) |
|  | P | 0.809 | 0.000224 | 0.483 | 0.620 | 0.0899 | 0.980 |
| PSA density [ng/ml/cc] | Coeff | -0.463 | 0.999 | -3.976 | -0.969 | 4.023*** | -2.009 |
|  | 95%CI | (-5.849 - 4.922) | (-0.478 - 2.476) | (-16.46 - 8.508) | (-11.05 - 9.109) | (1.052 - 6.995) | (-8.031 - 4.013) |
|  | P | 0.866 | 0.185 | 0.526 | 0.849 | 0.00848 | 0.510 |
| Positive DRE | Coeff | 0.363 | 1.084*** | -0.678 | -0.179 | -0.263 | -0.741 |
|  | 95%CI | (-0.798 - 1.524) | (0.417 - 1.751) | (-4.042 - 2.687) | (-2.323 - 1.965) | (-1.583 - 1.057) | (-2.243 - 0.762) |
|  | P | 0.540 | 0.00148 | 0.688 | 0.869 | 0.694 | 0.330 |
| PIRADS 4 lesion§ | Coeff | 1.531** | 1.345*** | -2.811 | 2.481** | 0.914* | 1.649** |
|  | 95%CI | (0.0388 - 3.023) | (0.823 - 1.867) | (-7.321 - 1.700) | (0.0597 - 4.902) | (-0.133 - 1.961) | (0.102 - 3.196) |
|  | P | 0.0444 | 5.53e-07 | 0.217 | 0.0447 | 0.0864 | 0.0370 |
| PIRADS 5 lesion§ | Coeff | 0.244 | 1.380*** | -5.985** | 2.018 | 2.035*** | 1.284 |
|  | 95%CI | (-1.644 - 2.133) | (0.574 - 2.186) | (-11.32 - -0.646) | (-1.153 - 5.188) | (0.549 - 3.521) | (-1.015 - 3.582) |
|  | P | 0.799 | 0.000823 | 0.0287 | 0.209 | 0.00776 | 0.270 |
| Posterior lesion §§ | Coeff | 0.0343 | 0.547** | 0.0139 | 0.266 | -0.302 | -0.0775 |
|  | 95%CI | (-1.183 - 1.251) | (0.0357 - 1.058) | (-3.445 - 3.473) | (-1.964 - 2.497) | (-1.286 - 0.681) | (-1.600 - 1.445) |
|  | P | 0.956 | 0.0361 | 0.994 | 0.813 | 0.543 | 0.920 |
| Poster+anterior lesion§§ | Coeff | 1.551 | 1.071* | 0.458 |  | 0.286 | 1.106 |
|  | 95%CI | (-4.888 - 7.989) | (-0.107 - 2.248) | (-9.202 - 10.12) |  | (-1.622 - 2.194) | (-1.554 - 3.766) |
|  | P | 0.636 | 0.0746 | 0.925 |  | 0.767 | 0.411 |
| Main target diameter [mm] | Coeff | -0.0866 | 0.0254 | -0.302* | -0.104 | -0.00146 | 0.0648 |
|  | 95%CI | (-0.202 - 0.0284) | (-0.0205 - 0.0713) | (-0.614 - 0.00919) | (-0.333 - 0.125) | (-0.0814 - 0.0785) | (-0.0738 - 0.203) |
|  | P | 0.140 | 0.277 | 0.0569 | 0.370 | 0.971 | 0.356 |
| ASA 2§§§ | Coeff | -0.0740 | 0.855** | -3.279* | -1.829* | -0.582 | 1.015 |
|  | 95%CI | (-1.217 - 1.069) | (0.158 - 1.551) | (-6.652 - 0.0948) | (-3.729 - 0.0704) | (-2.445 - 1.280) | (-1.491 - 3.522) |
|  | P | 0.899 | 0.0163 | 0.0566 | 0.0589 | 0.536 | 0.423 |
| ASA 3 §§§ | Coeff | -0.387 | 0.960 | -1.400 | -3.240 | -1.429 | -1.667 |
|  | 95%CI | (-2.669 - 1.895) | (-1.630 - 3.551) | (-8.252 - 5.452) | (-7.381 - 0.901) | (-6.506 - 3.649) | (-6.441 - 3.108) |
|  | P | 0.739 | 0.467 | 0.684 | 0.124 | 0.578 | 0.490 |
| ASA 4 §§§ | Coeff | 10.15* | 2.293 |  | 10.96** | 0.571 |  |
|  | 95%CI | (-0.853 - 21.16) | (-2.101 - 6.688) |  | (2.044 - 19.88) | (-4.506 - 5.649) |  |
|  | P | 0.0705 | 0.306 |  | 0.0166 | 0.824 |  |
| ECOG PS 1 §§§§ | Coeff | 0.0825 | -0.290 | -2.669 | -0.137 | -0.749 | 0.0444 |
|  | 95%CI | (-1.153 - 1.318) | (-0.985 - 0.404) | (-6.343 - 1.006) | (-2.232 - 1.958) | (-2.001 - 0.503) | (-1.932 - 2.020) |
|  | P | 0.896 | 0.412 | 0.151 | 0.897 | 0.238 | 0.965 |
| ECOG PS 2 §§§§ | Coeff | 0.126 | -0.0223 | -1.048 | -1.356 | -0.812 | 0.307 |
|  | 95%CI | (-1.436 - 1.689) | (-0.700 - 0.655) | (-5.498 - 3.403) | (-4.897 - 2.186) | (-2.051 - 0.428) | (-1.537 - 2.151) |
|  | P | 0.874 | 0.949 | 0.639 | 0.449 | 0.197 | 0.742 |
| ECOG PS 3 §§§§ | Coeff | -2.549 | 1.451 |  | -3.481 | 0.522 | -1.375 |
|  | 95%CI | (-8.119 - 3.021) | (-2.949 - 5.851) |  | (-10.20 - 3.239) | (-4.295 - 5.338) | (-8.792 - 6.042) |
|  | P | 0.369 | 0.518 |  | 0.306 | 0.830 | 0.714 |
| Anxiety NRS (0-10) | Coeff | 0.0966 | 0.0406 | 0.0339 | 0.00310 | 0.311** | -0.0656 |
|  | 95%CI | (-0.0776 - 0.271) | (-0.126 - 0.208) | (-0.550 - 0.618) | (-0.276 - 0.282) | (0.0133 - 0.608) | (-0.520 - 0.389) |
|  | P | 0.276 | 0.633 | 0.908 | 0.982 | 0.0408 | 0.775 |
| Total biopsy cores | Coeff | 0.810*** | 1.279*** | 1.752*** | 0.605** | 1.272*** | 1.208*** |
|  | 95%CI | (0.451 - 1.168) | (1.121 - 1.436) | (0.726 - 2.778) | (0.00275 - 1.207) | (0.970 - 1.573) | (0.799 - 1.617) |
|  | P | 1.17e-05 | 0 | 0.00115 | 0.0490 | 0 | 6.31e-08 |
| Number of targets | Coeff | 3.352*** | 2.579*** | 6.099*** | 2.150* | 2.500*** | 2.409*** |
|  | 95%CI | (2.205 - 4.499) | (2.268 - 2.890) | (2.632 - 9.566) | (-0.092 - 4.392) | (1.908 - 3.092) | (1.603 - 3.215) |
|  | P | 0.000 | 0.000 | 0.001 | 0.060 | 0.000 | 0.000 |
| Observations |  | 402 | 608 | 62 | 94 | 100 | 100 |
