# Supplementary material for: Impact of operator expertise on transperineal free-hand mpMRI-fusion-targeted biopsies under local anaesthesia for prostate cancer diagnosis: a multicenter prospective learning curve
Source: World J Urol. 2023 Oct 12;41(12):3867–76. doi: 10.1007/s00345-023-04642-2 (PMC10693515; doi:10.1007/s00345-023-04642-2)
Supplement: Supplementary file 6 — Supplementary file6 (DOCX 32 KB) [file 345_2023_4642_MOESM6_ESM.docx]

**Supplementary Table 3.** Univariable logistic regression analysis for clinically significant prostate cancer detection reate on target biopsy (csCDR-T), per Centre and per Operator (Op). Significant coefficients are highlighted in green. NRS = numerical rating scale; BMI = body mass index; DRE = digital rectal examination; PIRADS = Prostate Index Reporting and Data System score; ECOG PS = Eastern Cooperative Oncology Group Performance Status; § = reference is PIRADS 3; §§ = reference is anterior target; §§§ = reference is ECOG PS 0. *, omitted due to quasi-separation; ** Firth regression applied.

| csCDR-T  univariable regression | | Centre 1 | Centre 2 | Op1 | | Op2 | Op3 | Op4 |
| --- | --- | --- | --- | --- | --- | --- | --- | --- |
| Age | OR | 0,079 | 0,041 | 0,056 | | 0,087 | -0,003 | 0,053 |
|  | 95%CI | 0,050 - 0,109 | 0,009 - 0,073 | -0,024 - 0,135 | | 0,021 - 0,154 | -0,103 - 0,097 | -0,040 - 0,145 |
|  | *p* | *0,0001* | *0,012* | *0,169* | | *0,01* | *0,95* | *0,263* |
| Centre experience | OR | 0,001 | 0,002 |  | |  |  |  |
|  | 95%CI | -0,001 - 0,003 | 0,001 - 0,004 |  | |  |  |  |
|  | *p* | *0,203* | *0,001* |  | |  |  |  |
| Operator experience | OR |  |  | 0,002 | | 0,008 | -0,01 | 0,008 |
|  | 95%CI |  |  | -0,025 - 0,029 | | -0,008 - 0,023 | -0,039 - 0,019 | -0,013 - 0,028 |
|  | *p* |  |  | *0,876* | | *0,327* | *0,505* | *0,47* |
| Pain NRS (0-10) | OR | -0,076 | 0,058 | -0,174 | | 0,003 | -0,175 | -0,004 |
|  | 95%CI | -0,156 - 0,004 | -0,060 - 0,176 | -0,396 - 0,047 | | -0,164 - 0,169 | -0,742 - 0,393 | -0,320 - 0,313 |
|  | *p* | *0,063* | *0,335* | *0,123* | | *0,976* | *0,546* | *0,982* |
| Positive family history | OR | -0,486 | 0,873 | -0,644 | | -0,679 | * | 2.546 |
|  | 95%CI | -1,085 - 0,113 | -0,101 - 1,848 | -2,505 - 1,218 | | -1,908 - 0,549 |  | 0,640 - 4,451 |
|  | *p* | *0,112* | *0,079* | *0,498* | | *0,278* |  | *0,009* |
| Charlson score | OR | 0,412 | 0,166 | 0,12 | | 0,439 | -1.119 | -0,7 |
|  | 95%CI | 0,219 - 0,605 | -0,103 - 0,435 | -0,385 - 0,624 | | 0,031 - 0,846 | -2,512 - 0,274 | -1,764 - 0,365 |
|  | *p* | *0,0001* | *0,227* | *0,642* | | *0,035* | *0,115* | *0,198* |
| BMI | OR | -0,007 | 0,012 | 0,048 | | 0,082 | -0,172 | -0,049 |
|  | 95%CI | -0,068 - 0,054 | -0,069 - 0,093 | -0,103 - 0,198 | | -0,044 - 0,209 | -0,498 - 0,154 | -0,249 - 0,151 |
|  | *p* | *0,824* | *0,776* | *0,534* | | *0,2* | *0,301* | *0,633* |
| PSA | OR | 0,053 | 0,118 | -0,006 | | 0,152 | 0,12 | 0,191 |
|  | 95%CI | -0,002 - 0,107 | 0,069 - 0,168 | -0,124 - 0,113 | | 0,018 - 0,287 | -0,058 - 0,298 | 0,035 - 0,346 |
|  | *p* | *0,057* | *0,0001* | *0,927* | | *0,027* | *0,186* | *0,016* |
| Prostate volume | OR | -0,025 | -0,037 | -0,051 | | -0,009 | -0,055 | -0,038 |
|  | 95%CI | -0,035 - -0,016 | -0,051 - -0,024 | -0,083 - -0,019 | | -0,023 - 0,005 | -0,115 - 0,005 | -0,080 - 0,004 |
|  | *p* | *0,0001* | *0,0001* | *0,002* | | *0,213* | *0,074* | *0,077* |
| PSA density | OR | 6.919 | 5.209 | 3.953 | | 6.467 | 6.123 | 8.662 |
|  | 95%CI | 4,459 - 9,378 | 3,862 - 6,557 | -0,621 - 8,527 | | 1,605 - 11,330 | 1,885 - 10,361 | 3,884 - 13,439 |
|  | *p* | *0,0001* | *0,0001* | *0,09* | | *0,009* | *0,005* | *0,0001* |
| Positive DRE | OR | 1.105 | 1.013 | 1.631 | | 1.276 | 1.136 | 2.112 |
|  | 95%CI | 0,676 - 1,533 | 0,502 - 1,523 | 0,511 - 2,752 | | 0,333 - 2,218 | -0,659 - 2,932 | 0,741 - 3,483 |
|  | *p* | *0,0001* | *0,0001* | *0,004* | | *0,008* | *0,215* | *0,003* |
| PI-RADS 4 lesion § | OR | 1.170 | 1.779 | 1.486 | | 1.366 | 2.031 | -1.235 |
|  | 95%CI | 0,534 - 1,807 | 1,145 - 2,414 | -0,183 - 3,156 | | 0,030 - 2,703 | -0,280 - 4,343 | -2,582 - 0,113 |
|  | *p* | *0,0001* | *0,0001* | *0,081* | | *0,045* | *0,085* | *0,073* |
| PI-RADS 5 lesion § | OR | 2.465 | 2.515 | 3.871 | | 2.079 | 2.607 | 3.827** |
|  | 95%CI | 1,661 - 3,269 | 1,774 - 3,257 | 1,309 - 6,433 | | 0,471 - 3,688 | 0,106 - 5,107 | 0,837 - 6,818 |
|  | *p* | *0,0001* | *0,0001* | *0,003* | | *0,011* | *0,041* | *0,012*** |
| Posterior target §§ | OR | -0,315 | 0,3 | 0,182 | | 0,258 | 1.302 | 0,515 |
|  | 95%CI | -0,745 - 0,116 | -0,173 - 0,772 | -0,834 - 1,199 | | -0,722 - 1,238 | -1,000 - 3,603 | -0,764 - 1,795 |
|  | *p* | *0,152* | *0,214* | *0,725* | | *0,605* | *0,268* | *0,43* |
| Anterior + Posterior target §§ | OR | -0,761 | 1.122 | * | | * | 2.976 | 0,118 |
|  | 95%CI | -3,188 - 1,666 | 0,267 - 1,977 | | |  | 0,405 - 5,546 | -2,204 - 2,439 |
|  | *p* | *0,539* | *0,01* |  | |  | *0,023* | *0,921* |
| Target diameter [mm] | OR | 0,158 | 0,058 | 0,093 | | 0,222 | 0,108 | 0,062 |
|  | 95%CI | 0,108 - 0,208 | 0,022 - 0,095 | -0,012 - 0,198 | | 0,102 - 0,342 | -0,008 - 0,224 | -0,042 - 0,166 |
|  | *p* | *0,0001* | *0,002* | *0,083* | | *0,0001* | *0,067* | *0,243* |
| ASA score 2 | OR | 0,298 | -0,437 | -0,163 | | 0,326 | 0,131** | 0,134 |
|  | 95%CI | -0,111 - 0,707 | -1,011 - 0,138 | -1,183 - 0,858 | | -0,555 - 1,207 | -2,840 - 3,103 | -2,039 - 2,306 |
|  | *p* | *0,153* | *0,136* | *0,755* | | *0,468* | *0,931*** | *0,904* |
| ASA score 3 | OR | 1.170 | 1.414 | 0,875 | | * | 1.609** | 1.386 |
|  | 95%CI | 0,294 - 2,046 | -0,267 - 3,095 | -1,512 - 3,263 |  | | -2,685 - 5,904 | -1,789 - 4,562 |
|  | *p* | *0,009* | *0,099* | *0,472* | |  | *0,463* | *0,392* |
| ECOG PS 1 §§§ | OR | 0,312 | -0,192 | -0,295 | | 0,56 | 0,229** | 0,036 |
|  | 95%CI | -0,129 - 0,752 | -0,830 - 0,446 | -1,392 - 0,803 | | -0,354 - 1,473 | -2,230 - 2,688 | -1,566 - 1,638 |
|  | *p* | *0,166* | *0,556* | *0,599* | | *0,23* | *0,855* | *0,965* |
| ECOG PS 2§§§ | OR | 0,807 | 0,163 | 0,486 | | 1.910 | 0,606 | 0,1 |
|  | 95%CI | 0,241 - 1,373 | -0,433 - 0,759 | -0,920 - 1,891 | | 0,204 - 3,615 | -1,718 - 2,931 | -1,385 - 1,585 |
|  | *p* | *0,005* | *0,592* | *0,498* | | *0,028* | *0,609* | *0,895* |
| ECOG PS3 §§§ | OR | 1.513 | * | * | | 0,811 | 1.609** | 0,717** |
|  | 95%CI | -0,769 - 3,794 |  |  | | -2,023 - 3,645 | -1,993 - 5,212 | -2,677 - 4,111 |
|  | *p* | *0,194* |  |  | | *0,575* | *0,381*** | *0,679*** |
| Anxiety NRS (0-10) | OR | -0,014 | -0,055 | -0,008 | | -0,003 | 0,294 | 0,031 |
|  | 95%CI | -0,076 - 0,048 | -0,210 - 0,101 | -0,182 - 0,166 | | -0,122 - 0,116 | -0,136 - 0,724 | -0,328 - 0,390 |
|  | *p* | *0,651* | *0,491* | *0,93* | | *0,959* | *0,181* | *0,864* |
| Total biopsy cores | OR | 0,123 | -0,097 | 0,055 | | 0,2 | 0,408 | -0,196 |
|  | 95%CI | -0,011 - 0,258 | -0,273 - 0,079 | -0,288 - 0,398 | | -0,090 - 0,489 | -0,222 - 1,038 | -0,594 - 0,202 |
|  | *p* | *0,072* | *0,278* | *0,754* | | *0,177* | *0,204* | *0,334* |
| Double MRI target | OR | 0,251 | -0,121 | -0,189 | | 0,811 | 0,839 | -0,758 |
|  | 95%CI | -0,242 - 0,744 | -0,612 - 0,371 | -1,542 - 1,163 | | -0,280 - 1,902 | -1,000 - 2,678 | -2,119 - 0,603 |
|  | *p* | *0,318* | *0,63* | *0,784* | | *0,145* | *0,371* | *0,275* |
| Triple MRI target | OR | 1.142 | -0,551 | * | | * | 1.686 | -0,613 |
|  | 95%CI | -0,512 - 2,797 | -1,440 - 0,339 | | |  | -0,883 - 4,256 | -2,106 - 0,880 |
|  | *p* | *0,176* | *0,225* |  | |  | *0,198* | *0,421* |
