# Supplementary material for: Impact of operator expertise on transperineal free-hand mpMRI-fusion-targeted biopsies under local anaesthesia for prostate cancer diagnosis: a multicenter prospective learning curve
Source: World J Urol. 2023 Oct 12;41(12):3867–76. doi: 10.1007/s00345-023-04642-2 (PMC10693515; doi:10.1007/s00345-023-04642-2)
Supplement: Supplementary file 7 — Supplementary file7 (DOCX 39 KB) [file 345_2023_4642_MOESM7_ESM.docx]

**Supplementary Table 4.** Consecutive patients group characteristics in Centre 1 (A) and Centre 2 (B). Patients groups have 50 consecutive patients each. Learning curve variables are tested for a trend among groups (Cochrane-Armitage test or Jonckheere-Terpstra test, *p trend*). Patient variables are tested for differences among groups (Pearson Chi-square or Kruskal-Wallis test, *p diff*). csPCa = clinically significant prostate cancer; BMI = body mass index; DRE = digital rectal examination; PIRADS = Prostate Index Reporting and Data System score; A = anterior; P = posterior; A+P = anterior and posterior; ISUP GG = International Society of Urological Pathology grade group; ECOG PS = Eastern Cooperative Oncology Group Performance Status; NRS = numerical rating scale. Significant p values are highlighted in green.

| A. Centre 1 | Patient group | 1 - 50 | 51 - 100 | 101 - 150 | 151 - 200 | 201 - 250 | 251 - 300 | 301 - 350 | 351 - 400 | 401 - 450 | 451 - 500 | 501 - 550 | 551 - 600 | . |
| --- | --- | --- | --- | --- | --- | --- | --- | --- | --- | --- | --- | --- | --- | --- |
| Learning variable | | **N (%) or median (IQR)** | | | | | | | | | | | | **P trend** |
| csPCa on target | + | 22 (44) | 25 (50) | 19 (38) | 19 (38) | 23 (46) | 20 (40) | 32 (64) | 23 (46) | . | . | . | , | 0,201 |
| csPCa on mapping | + | 20 (40) | 19 (38) | 17 (34) | 18 (36) | 15 (30) | 18 (36) | 33 (66) | 19 (38) | . | . | . | , | 0,062 |
| Total time | min | 20 (17-25,3) | 16 (13-21,3) | 16,5 (14-20) | 18,5 (14,8-22) | 20 (16-23) | 17,5 (15-20) | 16 (14-20,3) | 19 (16,8-24) | . | . | . | , | 0,717 |
| Patient variable | | **N (%) or median (IQR)** | | | | | | | | | | | | **P diff** |
| Age | Years | 68 (61,8-72) | 67,5 (62-72,3) | 68,5 (60-73) | 66,5 (60,8-72) | 69 (65,8-75) | 69 (60,8-74) | 66 (60-72) | 68,5 (62-75) | . | . | . | . | 0,516 |
| BMI | kg/m2 | 25 (23,1-27,8) | 25,9 (23,9-28,7) | 25 (23,5-27,9) | 24,5 (23,2-26,4) | 24,9 (22,8-27,2) | 25,8 (24-27,5) | 24,7 (22,9-27,2) | 26,1 (23,7-27,7) | . | . | . | . | 0,075 |
| Family history | + | 4 (8) | 7 (14) | 6 (12) | 7 (14) | 10 (20) | 5 (10) | 9 (18) | 5 (10) | . | . | . | . | 0,636 |
| DRE | + | 21 (42) | 20 (40) | 18 (36) | 21 (42) | 14 (28) | 12 (24) | 17 (34) | 10 (20) | . | . | . | . | 0,132 |
| PSA | ng/ml | 6,7 (4,9-8,4) | 6,3 (5,2-10,1) | 6,1 (4,5-10,1) | 6,1 (5-7,2) | 6,8 (5-8,4) | 6,1 (4,8-8,7) | 6,9 (4,9-8,7) | 5,5 (4,1-7,1) | . | . | . | . | 0,269 |
| Prostate volume | cc | 44 (32-57) | 50 (33-64) | 48 (41-63) | 48 (33-61) | 43 (31-62) | 50 (39-67) | 39 (29-58) | 44 (32-64) | . | . | . | . | 0,299 |
| PSA density | ng/ml/cc | 0,14 (0,1-0,22) | 0,16 (0,1-0,22) | 0,13 (0,09-0,22) | 0,13 (0,08-0,19) | 0,15 (0,1-0,25) | 0,11 (0,09-0,17) | 0,15 (0,11-0,24) | 0,13 (0,06-0,17) | . | . | . | . | 0,09 |
| PIRADS score (target 1) | 3 | 7 (16,7) | 7 (14) | 9 (18,8) | 6 (13,3) | 7 (14,6) | 13 (26,5) | 10 (20) | 8 (16) | . | . | . | . | 0,76 |
|  | 4 | 27 (64,3) | 34 (68) | 29 (60,4) | 29 (64,4) | 36 (75) | 30 (61,2) | 29 (58) | 36 (72) | . | . | . | . | . |
|  | 5 | 8 (19) | 9 (18) | 10 (20,8) | 10 (22,2) | 5 (10,4) | 6 (12,2) | 11 (22) | 6 (12) | . | . | . | . | . |
| Target 1 maximum diameter | mm | 10 (6-13) | 10 (6,3-14) | 10 (7-15) | 8 (6,3-11) | 8,5 (6-11,3) | 8 (6-12) | 10,5 (7-14) | 9 (7-12,5) | . | . | . | . | 0,296 |
| Target location | A | 15 (30) | 13 (26) | 12 (24) | 8 (16) | 23 (46) | 16 (32) | 13 (26) | 14 (28) | . | . | . | . | 0,063 |
|  | P | 35 (70) | 37 (74) | 38 (76) | 41 (82) | 27 (54) | 32 (64) | 37 (74) | 36 (72) | . | . | . | . | . |
|  | A+P | 0 (0) | 0 (0) | 0 (0) | 1 (2) | 0 (0) | 2 (4) | 0 (0) | 0 (0) | . | . | . | . | . |
| ISUP GG on target | No cancer | 27 (54) | 24 (48) | 31 (62) | 30 (60) | 26 (52) | 30 (60) | 17 (34) | 24 (48) | . | . | . | . | 0,215 |
|  | 1 | 1 (2) | 1 (2) | 0 (0) | 1 (2) | 1 (2) | 0 (0) | 1 (2) | 3 (6) | . | . | . | . | . |
|  | 2 | 13 (26) | 12 (24) | 12 (24) | 13 (26) | 14 (28) | 7 (14) | 13 (26) | 15 (30) | . | . | . | . | . |
|  | 3 | 6 (12) | 8 (16) | 6 (12) | 5 (10) | 7 (14) | 11 (22) | 18 (36) | 8 (16) | . | . | . | . | . |
|  | 4 | 2 (0,04) | 4 (0,08) | 1 (0,02) | 1 (0,02) | 2 (0,04) | 2 (0,04) | 1 (0,02) | 0 (0) | . | . | . | . | . |
|  | 5 | 1 (2) | 1 (2) | 0 (0) | 0 (0) | 0 (0) | 0 (0) | 0 (0) | 0 (0) | . | . | . | . | . |
| MRI targets | 1 | 36 (72) | 33 (66) | 43 (86) | 48 (96) | 40 (80) | 42 (84) | 35 (70) | 38 (76) | . | . | . | . | 0,016 |
|  | 2 | 14 (28) | 14 (28) | 7 (14) | 1 (2) | 9 (18) | 7 (14) | 14 (28) | 12 (24) | . | . | . | . | . |
|  | 3 | 0 (0) | 3 (6) | 0 (0) | 1 (2) | 1 (2) | 1 (2) | 1 (2) | 0 (0) | . | . | . | . | . |
| Pain NRS | 0-10 | 5 (3-7) | 5 (4-7) | 4 (2-6) | 3 (2,8-6,3) | 4 (3-6) | 4 (2-5,3) | 5 (3-7) | 6 (3-7,3) | . | . | . | . | 0,003 |
| Anxiety NRS | 0-10 | 3 (0-6) | 5 (1-7,3) | 3 (0-6,3) | 3 (1-5) | 4 (1-7) | 2 (1-5) | 5 (2-8) | 3 (1-5,3) | . | . | . | . | 0,028 |

| B. Centre 2 | Patient group | 1 - 50 | 51 - 100 | 101 - 150 | 151 - 200 | 201 - 250 | 251 - 300 | 301 - 350 | 351 - 400 | 401 - 450 | 451 - 500 | 501 - 550 | 551 - 600 | . |
| --- | --- | --- | --- | --- | --- | --- | --- | --- | --- | --- | --- | --- | --- | --- |
| Learning variable | | **N (%) or median (IQR)** | | | | | | | | | | | | **P trend** |
| csPCa on target | + | 5 (10) | 8 (16) | 5 (10) | 2 (4) | 7 (14) | 6 (12) | 3 (6) | 5 (10) | 6 (12) | 11 (22) | 23 (46) | 7 (14) | 0,001 |
| csPCa on mapping | + | 6 (12) | 9 (18) | 7 (14) | 8 (16) | 10 (20) | 12 (24) | 7 (14) | 9 (18) | 9 (18) | 15 (30) | 20 (40) | 10 (20) | 0,002 |
| Total_time | min | 18 (15,8-20,3) | 16 (15-19) | 14,5 (12,8-16) | 13 (12-15) | 13 (12-15) | 14 (12-16) | 13 (12-15) | 12 (11-14) | 12 (10-13) | 12 (10-14) | 12,5 (11,8-14,3) | 13 (12-14) | 0,001 |
| Patient variable | | **N (%) or median (IQR)** | | | | | | | | | | | | **P diff** |
| Age | Years | 68 (62,8-72,3) | 68 (63-73) | 66 (59,8-72,3) | 68,5 (60-74) | 65,5 (59-71) | 66,5 (59,8-73) | 66 (61,8-70) | 67 (59,5-73) | 67 (60,8-72,3) | 66 (63-72) | 68,5 (64-72,3) | 67 (63-72) | 0,794 |
| BMI | kg/m2 | 23,5 (21,6-25,8) | 24,2 (21,4-26,4) | 24,1 (20,8-25,9) | 24,2 (22,4-26) | 24,4 (22,7-26,1) | 24,6 (23,2-25,9) | 24,2 (22,7-26) | 24,9 (22,9-26) | 24,5 (22,1-27,4) | 24,8 (23,1-26,6) | 24 (22,5-26,5) | 25 (22,9-26,7) | 0,189 |
| Family history | + | 2 (4) | 2 (4) | 2 (4) | 1 (2) | 1 (2) | 1 (2) | 3 (6) | 3 (6) | 1 (2) | 1 (2) | 0 (0) | 3 (6) | 0,829 |
| DRE | + | 15 (30) | 20 (40) | 10 (20) | 4 (8) | 11 (22) | 6 (12) | 7 (14) | 5 (10) | 4 (8) | 5 (10) | 9 (18) | 3 (6) | 0,001 |
| PSA | ng/ml | 6,7 (4,8-10) | 6,6 (5,3-8,8) | 7,8 (5,2-11,2) | 8,3 (6-10,9) | 8,9 (6,1-14) | 9,2 (5,3-11,8) | 9,2 (5,9-13,3) | 6,6 (5,2-9,6) | 8,6 (5,2-11,9) | 8,6 (6-11) | 7,8 (4,9-12,7) | 8,5 (5,1-10,3) | 0,083 |
| Prost_vol | cc | 43 (27-53) | 39 (27-63) | 41 (27-62) | 52 (28-88) | 38 (23-53) | 43 (31-60) | 46 (28-62) | 53 (34-72) | 49 (36-61) | 44 (33-61) | 45 (28-64) | 51 (39-75) | 0,066 |
| PSAd | ng/ml/cc | 0,16 (0,11-0,24) | 0,18 (0,12-0,25) | 0,19 (0,12-0,3) | 0,16 (0,1-0,26) | 0,23 (0,17-0,31) | 0,18 (0,12-0,33) | 0,19 (0,12-0,3) | 0,13 (0,09-0,22) | 0,15 (0,12-0,26) | 0,18 (0,11-0,27) | 0,17 (0,1-0,29) | 0,14 (0,1-0,22) | 0,011 |
| PIRADS target 1 | 3 | 16 (32) | 18 (36) | 31 (62) | 29 (58) | 28 (56) | 23 (46) | 23 (46) | 28 (56) | 23 (46) | 20 (40) | 21 (42) | 31 (62) | 0,225 |
|  | 4 | 26 (52) | 26 (52) | 15 (30) | 15 (30) | 16 (32) | 19 (38) | 22 (44) | 18 (36) | 22 (44) | 23 (46) | 25 (50) | 14 (28) | . |
|  | 5 | 8 (16) | 6 (12) | 4 (8) | 6 (12) | 6 (12) | 8 (16) | 5 (10) | 4 (8) | 5 (10) | 7 (14) | 4 (8) | 5 (10) | . |
| Target 1 diameter | mm | 12 (8,8-15,5) | 11,5 (7,8-14,3) | 10 (8-14,3) | 11 (8-18) | 10 (8-12,3) | 10 (7,8-14) | 9,5 (7-13) | 9 (6-12) | 8 (6,8-12,3) | 9 (6-13) | 10 (7,8-12) | 10 (8-14) | 0,03 |
| Target location | A | 21 (42) | 18 (36) | 22 (44) | 27 (54) | 25 (50) | 23 (46) | 26 (52) | 28 (56) | 33 (66) | 33 (66) | 15 (30) | 19 (38) | 0,018 |
|  | P | 25 (50) | 27 (54) | 26 (52) | 19 (38) | 21 (42) | 25 (50) | 23 (46) | 22 (44) | 16 (32) | 15 (30) | 32 (64) | 30 (60) | . |
|  | B | 4 (8) | 5 (10) | 2 (4) | 4 (8) | 4 (8) | 2 (4) | 1 (2) | 0 (0) | 1 (2) | 2 (4) | 3 (6) | 1 (2) | . |
| ISUP GG su target | No cancer | 40 (80) | 35 (70) | 41 (82) | 37 (74) | 35 (70) | 32 (64) | 38 (76) | 38 (76) | 37 (74) | 34 (68) | 25 (50) | 34 (68) | 0,001 |
|  | 1 | 5 (10) | 7 (14) | 4 (8) | 11 (22) | 8 (16) | 12 (24) | 9 (18) | 7 (14) | 7 (14) | 5 (10) | 2 (4) | 9 (18) | . |
|  | 2 | 3 (6) | 1 (2) | 3 (6) | 2 (4) | 2 (4) | 2 (4) | 2 (4) | 2 (4) | 3 (6) | 3 (6) | 9 (18) | 3 (6) | . |
|  | 3 | 2 (4) | 0 (0) | 0 (0) | 0 (0) | 4 (8) | 2 (4) | 1 (2) | 1 (2) | 0 (0) | 5 (10) | 10 (20) | 1 (2) | . |
|  | 4 | 0 (0) | 6 (12) | 0 (0) | 0 (0) | 1 (2) | 2 (4) | 0 (0) | 2 (4) | 3 (6) | 3 (6) | 4 (8) | 3 (6) | . |
|  | 5 | 0 (0) | 1 (2) | 2 (4) | 0 (0) | 0 (0) | 0 (0) | 0 (0) | 0 (0) | 0 (0) | 0 (0) | 0 (0) | 0 (0) | . |
| MRI targets | 1 | 17 (34) | 16 (32) | 22 (44) | 31 (62) | 34 (68) | 27 (54) | 27 (54) | 27 (54) | 31 (62) | 37 (74) | 37 (74) | 43 (86) | 0,001 |
|  | 2 | 18 (36) | 22 (44) | 24 (48) | 17 (34) | 15 (30) | 17 (34) | 14 (28) | 21 (42) | 16 (32) | 12 (24) | 12 (24) | 3 (6) | . |
|  | 3 | 15 (30) | 12 (24) | 4 (8) | 2 (4) | 1 (2) | 6 (12) | 9 (18) | 2 (4) | 3 (6) | 1 (2) | 1 (2) | 4 (8) | . |
| Pain NRS | 0-10 | 4 (4-6) | 4 (3-5,3) | 4 (3-5) | 4 (3-6) | 4,5 (3-7) | 4 (3-4,3) | 4 (3-6) | 4 (3-6,3) | 5 (3-7) | 4 (3-6) | 4 (4-7) | 4 (3-5) | 0,057 |
| Anxiety NRS | 0-10 | 3 (2-3) | 3 (2-3) | 3 (3-3) | 3 (2-3) | 3 (1-3) | 3 (2-3) | 3 (2-3) | 3 (2-3) | 3 (3-3) | 3 (2-3) | 3 (2-3) | 3 (2,8-3) | 0,42 |
