# Supplementary material for: Impact of operator expertise on transperineal free-hand mpMRI-fusion-targeted biopsies under local anaesthesia for prostate cancer diagnosis: a multicenter prospective learning curve
Source: World J Urol. 2023 Oct 12;41(12):3867–76. doi: 10.1007/s00345-023-04642-2 (PMC10693515; doi:10.1007/s00345-023-04642-2)
Supplement: Supplementary file 8 — Supplementary file8 (DOCX 39 KB) [file 345_2023_4642_MOESM8_ESM.docx]

**Supplementary Table 5.** Consecutive patients group characteristics in 4 operators (A, B, C, D). Patient groups have 16 patients each; the first 6 groups are considered when available (maximum 96 patients per operator). Patient variables are tested for differences among groups (Pearson Chi-square or Kruskal-Wallis test, *p diff*). Learning curve variables are tested for a trend among groups (Cochrane-Armitage test or Jonckheere-Terpstra test, *p trend*). csPCa = clinically significant prostate cancer; BMI = body mass index; DRE = digital rectal examination; PIRADS = Prostate Index Reporting and Data System score; A = anterior; P = posterior; A+P = anterior and posterior; NRS = numerical rating scale. Significant p values are highlighted in green.

| A. Operator 1 | Patient groups | 1 - 16 | 17 - 32 | 33 - 48 | 49 - 64 | 65 - 80 | 81 - 96 | . |
| --- | --- | --- | --- | --- | --- | --- | --- | --- |
| Learning variable | | **N (%) or median (IQR)** | | | | | | **P trend** |
| csPCa on target | + | 8 (50) | 9 (56,3) | 9 (56,3) | 9 (56,3) | . | . | 0,736 |
| csPCa mapping | + | 7 (43,8) | 7 (43,8) | 8 (50) | 10 (62,5) | . | . | 0,264 |
| Total time | min | 23,5 (20-27,5) | 17,5 (16-27) | 18,5 (16-20,8) | 18 (14-21,5) | . | . | 0,002 |
| Patient variable | | **N (%) or median (IQR)** | | | | | | **P diff** |
| Age | years | 70 (68-76,8) | 68,5 (61,3-74,5) | 66 (62,5-72) | 69 (66-72) | . | . | 0,192 |
| BMI | kg/m2 | 24,7 (23-26,1) | 26,4 (24,5-30) | 24,8 (21,1-27,1) | 25,3 (23,7-29,4) | . | . | 0,084 |
| Family history | + | 1 (6,3) | 1 (6,3) | 3 (18,8) | 0 (0) | . | . | 0,249 |
| DRE | + | 8 (50) | 4 (25) | 6 (37,5) | 8 (50) | . | . | 0,415 |
| PSA | ng/ml | 6,4 (4,2-9) | 7,5 (4,9-8,9) | 6,2 (5,1-7,9) | 6,8 (3,5-8,5) | . | . | 0,984 |
| Prostate volume | cc | 54 (39-61) | 51 (36-68) | 41 (30-55) | 35 (25-49) | . | . | 0,102 |
| PSA density | ng/ml/cc | 0,12 (0,07-0,26) | 0,15 (0,09-0,18) | 0,15 (0,09-0,25) | 0,14 (0,08-0,24) | . | . | 0,92 |
| PIRADS score (target 1) | 3 | 0 (0) | 4 (26,7) | 3 (18,8) | 3 (18,8) | . | . | 0,287 |
|  | 4 | 14 (87,5) | 8 (53,3) | 8 (50) | 10 (62,5) | . | . | . |
|  | 5 | 2 (12,5) | 3 (20) | 5 (31,3) | 3 (18,8) | . | . | . |
| Target 1 diameter (max) | mm | 10,5 (6,5-12,8) | 9 (6-19,3) | 8,5 (7,3-15) | 12 (8,5-14) | . | . | 0,886 |
| Target location | A | 8 (50) | 6 (37,5) | 5 (31,3) | 7 (43,8) | . | . | 0,322 |
|  | P | 8 (50) | 10 (62,5) | 9 (56,3) | 9 (56,3) | . | . | . |
|  | A+P | 0 (0) | 0 (0) | 2 (12,5) | 0 (0) | . | . | . |
| Pain NRS | 0-10 | 4,5 (2-6) | 4 (2,3-6,8) | 5 (4-6) | 5 (3,3-7) | . | . | 0,834 |
| Anxiety NRS | 0-10 | 4 (1-6,8) | 3,5 (1-7) | 5 (0,8-7,8) | 2,5 (1,3-4,8) | . | . | 0,59 |

| B. Operator 2 | Patient groups | 1 - 16 | 17 - 32 | 33 - 48 | 49 - 64 | 65 - 80 | 81 - 96 | . |
| --- | --- | --- | --- | --- | --- | --- | --- | --- |
| Learning variable | | **N (%) or median (IQR)** | | | | | | **P trend** |
| csPCa on target | + | 6 (37,5) | 7 (43,8) | 4 (25) | 4 (25) | 9 (56,3) | 7 (50) | 0,35 |
| csPCa mapping | + | 7 (43,8) | 5 (31,3) | 6 (37,5) | 2 (12,5) | 9 (56,3) | 7 (50) | 0,456 |
| Total time | min | 19 (16-24,5) | 16,5 (13,5-20,8) | 17,5 (14,3-22) | 15,5 (14-18,5) | 17 (14-18) | 14 (12,5-16,3) | 0,001 |
| Patient variable | | **N (%) or median (IQR)** | | | | | | **P diff** |
| Age | years | 66 (63-67,8) | 71 (61,5-75,5) | 64,5 (60,5-69) | 62,5 (55,5-69) | 68 (64-70,8) | 66,5 (60,8-74,3) | 0,311 |
| BMI | kg/m2 | 24,7 (23,4-27) | 24,9 (23-28,1) | 25 (23,2-26,5) | 24,5 (23,2-26,3) | 25,8 (22,9-28,4) | 23,8 (22,5-26,2) | 0,747 |
| Family history | + | 4 (25) | 2 (12,5) | 1 (6,3) | 5 (31,3) | 2 (12,5) | 1 (7,1) | 0,308 |
| DRE | + | 7 (43,8) | 6 (37,5) | 5 (31,3) | 3 (18,8) | 3 (18,8) | 2 (14,3) | 0,361 |
| PSA | ng/ml | 5,7 (4,1-7,3) | 7,3 (4,6-11,6) | 5 (4,4-6,8) | 7,3 (6,5-8,8) | 5,6 (4,8-8,9) | 7,5 (5,6-10,3) | 0,044 |
| Prostate volume | cc | 47 (31-54) | 61 (45-85) | 46 (33-79) | 40 (24-60) | 41 (33-79) | 57 (35-73) | 0,358 |
| PSA density | ng/ml/cc | 0,13 (0,08-0,2) | 0,13 (0,07-0,24) | 0,1 (0,06-0,15) | 0,2 (0,13-0,26) | 0,12 (0,07-0,19) | 0,15 (0,1-0,24) | 0,132 |
| PIRADS score (target 1) | 3 | 1 (6,3) | 2 (12,5) | 3 (18,8) | 4 (26,7) | 4 (25) | 5 (35,7) | 0,19 |
|  | 4 | 14 (87,5) | 11 (68,8) | 7 (43,8) | 10 (66,7) | 10 (62,5) | 7 (50) | . |
|  | 5 | 1 (6,3) | 3 (18,8) | 6 (37,5) | 1 (6,7) | 2 (12,5) | 2 (14,3) | . |
| Target 1 diameter (max) | mm | 9,5 (5,3-13) | 10 (6-14,8) | 9 (6-15,8) | 8 (6-10,5) | 8 (7-11,8) | 9 (7,5-12,5) | 0,836 |
| Target location | A | 4 (25) | 3 (18,8) | 4 (25) | 6 (37,5) | 1 (6,3) | 5 (35,7) | 0,348 |
|  | P | 12 (75) | 13 (81,3) | 12 (75) | 10 (62,5) | 15 (93,8) | 9 (64,3) | . |
|  | A+P | 0 (0) | 0 (0) | 0 (0) | 0 (0) | 0 (0) | 0 (0) | . |
| Pain NRS | 0-10 | 6 (5-7,8) | 4,5 (2,3-6,8) | 3 (1-4) | 3 (2-6) | 2,5 (2-6,8) | 4 (2,8-5,3) | 0,014 |
| Anxiety NRS | 0-10 | 6,5 (1,8-7,8) | 4 (0-8,5) | 1 (0-4) | 2 (1-5) | 2 (1-7,8) | 5 (4,5-10) | 0,042 |

| C. Operator 3 | Patient groups | 1 - 16 | 17 - 32 | 33 - 48 | 49 - 64 | 65 - 80 | 81 - 96 | . |
| --- | --- | --- | --- | --- | --- | --- | --- | --- |
| Learning variable | | **N (%) or median (IQR)** | | | | | | **P trend** |
| csPCa on target | + | 2 (12,5) | 0 (0) | 2 (12,5) | 1 (6,3) | 0 (0) | 1 (6,3) | 0,459 |
| csPCa mapping | + | 4 (25) | 0 (0) | 3 (18,8) | 2 (12,5) | 2 (12,5) | 5 (31,3) | 0,423 |
| Total_time | min | 15,5 (14,3-17) | 13 (12-15,8) | 14,5 (12,3-16) | 13 (12-15) | 13 (12,3-14) | 13 (11,3-14) | 0,004 |
| Patient variable | | **N (%) or median (IQR)** | | | | | | **P diff** |
| Age | years | 69 (60,8-77) | 64,5 (61,3-71,8) | 68,5 (59-75,5) | 68,5 (62-72,8) | 68,5 (61,3-74,8) | 60 (57,3-71,8) | 0,686 |
| BMI | kg/m2 | 25,5 (20,6-26,7) | 24,2 (23,2-25,5) | 25 (22,3-25,8) | 23,5 (20,9-26,2) | 24,2 (23,1-26) | 24,2 (23,5-25,8) | 0,878 |
| Family history | + | 0 (0) | 0 (0) | 0 (0) | 0 (0) | 0 (0) | 0 (0) | 0,99 |
| DRE | + | 3 (18,75) | 3 (18,75) | 2 (12,5) | 1 (6,25) | 2 (12,5) | 4 (25) | 0,76 |
| PSA | ng/ml | 8,8 (6-11,3) | 9,2 (5,7-10,6) | 10 (5,3-11,6) | 7,9 (5,6-12,7) | 7,6 (5,5-10,7) | 8,3 (6,3-10) | 0,975 |
| Prostate volume | cc | 63 (29-82) | 48 (26-82) | 49 (20-85) | 50 (36-83) | 58 (28-98) | 43 (23-51) | 853 |
| PSA density | ng/ml/cc | 0,19 (0,09-0,25) | 0,19 (0,12-0,3) | 0,18 (0,1-0,45) | 0,16 (0,12-0,27) | 0,15 (0,08-0,23) | 0,2 (0,15-0,31) | 0,73 |
| PIRADS score (target 1) | 3 | 7 (43,8) | 14 (87,5) | 8 (50) | 9 (56,3) | 11 (68,8) | 10 (62,5) | 0,136 |
|  | 4 | 8 (50) | 1 (6,3) | 4 (25) | 4 (25) | 5 (31,3) | 4 (25) | . |
|  | 5 | 1 (6,3) | 1 (6,3) | 4 (25) | 3 (18,8) | 0 (0) | 2 (12,5) | . |
| Target 1 diameter (max) | mm | 10,5 (6-15) | 9,5 (7,3-11,8) | 15 (8,5-19,5) | 12 (8-19) | 9,5 (8,3-13,8) | 10 (7,3-13,8) | 0,453 |
| Target location | A | 8 (50) | 8 (50) | 5 (31,3) | 8 (50) | 10 (62,5) | 10 (62,5) | 0,475 |
|  | P | 8 (50) | 7 (43,8) | 8 (50) | 7 (43,8) | 6 (37,5) | 4 (25) | . |
|  | A+P | 0 (0) | 1 (6,3) | 3 (18,8) | 1 (6,3) | 0 (0) | 2 (12,5) | . |
| Pain NRS | 0-10 | 4 (3-5) | 4 (3-4,8) | 4 (3,3-5,8) | 4 (3-5) | 4 (3-6,8) | 4 (3,3-6,5) | 0,904 |
| Anxiety NRS | 0-10 | 3 (3-3) | 3 (3-5) | 3 (2-3) | 3 (1-3) | 3 (2,3-3) | 3 (2-3) | 0,038 |

| D. Operator 4 | Patient groups | 1 - 16 | 17 - 32 | 33 - 48 | 49 - 64 | 65 - 80 | 81 - 96 | . |
| --- | --- | --- | --- | --- | --- | --- | --- | --- |
| Learning variable | | **N (%) or median (IQR)** | | | | | | **P trend** |
| csPCa on target | + | 1 (6,3) | 3 (18,8) | 1 (6,3) | 3 (18,8) | 2 (12,5) | 3 (18,8) | 0,431 |
| csPCa mapping | + | 1 (6,3) | 3 (18,8) | 2 (12,5) | 3 (18,8) | 3 (18,8) | 2 (12,5) | 0,611 |
| Total time | min | 19 (17-24,8) | 17,5 (14,5-20) | 17 (15-18,8) | 16 (15-17) | 17 (14-19,8) | 16,5 (16-19) | 0,063 |
| Patient variable | | **N (%) or median (IQR)** | | | | | | **P diff** |
| Age | years | 66,5 (63,3-70,8) | 66 (60,5-70) | 70,5 (61,5-74) | 68 (61,5-74,3) | 68 (64,5-74,3) | 68 (63,8-73) | 0,667 |
| BMI | kg/m2 | 25,5 (22,8-27,4) | 23 (21,5-24,7) | 22,7 (20,9-23,7) | 23,1 (21-24,7) | 22,8 (20,9-26,4) | 24,8 (21,5-27,7) | 0,179 |
| Family history | + | 0 (0) | 2 (12,5) | 0 (0) | 0 (0) | 1 (6,3) | 1 (6,3) | 0,39 |
| DRE | + | 3 (18,75) | 9 (56,25) | 3 (18,75) | 4 (25) | 8 (50) | 7 (43,75) | 0,086 |
| PSA | ng/ml | 7,2 (3,9-11,7) | 6,7 (4,5-7,9) | 7,2 (5,8-9,2) | 6,2 (5,3-8,7) | 6,3 (4,9-8,6) | 6,5 (5,9-7,1) | 0,879 |
| Prostate volume | cc | 43 (32-54) | 30 (25-48) | 45 (33-56) | 43 (25-58) | 34 (27-45) | 39 (29-53) | 0,397 |
| PSA density | ng/ml/cc | 0,13 (0,08-0,3) | 0,17 (0,13-0,28) | 0,2 (0,11-0,25) | 0,18 (0,09-0,26) | 0,2 (0,14-0,28) | 0,15 (0,11-0,23) | 0,594 |
| PIRADS score (target 1) | 3 | 6 (37,5) | 4 (25) | 4 (25) | 8 (50) | 5 (31,3) | 6 (37,5) | 0,389 |
|  | 4 | 7 (43,8) | 9 (56,3) | 10 (62,5) | 4 (25) | 10 (62,5) | 10 (62,5) | . |
|  | 5 | 3 (18,8) | 3 (18,8) | 2 (12,5) | 4 (25) | 1 (6,3) | 0 (0) | . |
| Target 1 diameter (max) | mm | 11,5 (7,3-18) | 12 (10,3-14) | 10 (6,3-16,5) | 12,5 (8,3-14,8) | 10 (7,3-13,8) | 14 (9,3-15,8) | 0,568 |
| Target location | A | 7 (43,8) | 4 (25) | 9 (56,3) | 8 (50) | 4 (25) | 6 (37,5) | 0,413 |
|  | P | 6 (37,5) | 11 (68,8) | 7 (43,8) | 6 (37,5) | 11 (68,8) | 8 (50) | . |
|  | A+P | 3 (18,8) | 1 (6,3) | 0 (0) | 2 (12,5) | 1 (6,3) | 2 (12,5) | . |
| Pain NRS | 0-10 | 4 (4-6,8) | 4 (3,3-6) | 4 (4-7,3) | 4 (3-6) | 4 (4-5,5) | 4 (3,3-5,8) | 0,862 |
| Anxiety NRS | 0-10 | 3 (2-3) | 3 (2-3) | 3 (3-3,8) | 3 (2-3) | 3 (2-3) | 3 (3-3) | 0,428 |
