# Supplementary material for: Impact of operator expertise on transperineal free-hand mpMRI-fusion-targeted biopsies under local anaesthesia for prostate cancer diagnosis: a multicenter prospective learning curve
Source: World J Urol. 2023 Oct 12;41(12):3867–76. doi: 10.1007/s00345-023-04642-2 (PMC10693515; doi:10.1007/s00345-023-04642-2)
Supplement: Supplementary file 9 — Supplementary file9 (DOCX 25 KB) [file 345_2023_4642_MOESM9_ESM.docx]

Supplementary Table 6. Exact repetition of Table 2 (in A) and of Table 3 (in B) with the addition of 95% confidence intervals (95%CI) that had been removed from the main tables to help readability. See also legends of Table 2 and Table 3 for further information.

| **A. Biopsy time multivariable regression** | | **Centre 1** | Centre 2 | Operator 1 | Operator 2 | Operator 3 | Operator 4 |
| --- | --- | --- | --- | --- | --- | --- | --- |
| Centre experience | Coef. | -0.002 | -0.006 |  |  |  |  |
|  | 95%CI | (-0.007 - 0.003) | (-0.007 - -0.004) |  |  |  |  |
|  | p | 0.360 | 0.000 |  |  |  |  |
| Operator experience | Coef. |  |  | -0.150 | -0.044 | -0.010 | -0.049 |
|  | 95%CI |  |  | (-0.225 - -0.075) | (-0.079 - -0.008) | (-0.022 - 0.002) | (-0.069 - -0.029) |
|  | p |  |  | 0.000 | 0.016 | 0.109 | 0.000 |
| Age | Coef. | -0.023 | 0.035 | -0.307 | 0.100 | 0.026 | 0.033 |
|  | 95%CI | (-0.102 - 0.057) | (0.008 - 0.062) | (-0.524 - -0.091) | (-0.043 - 0.243) | (-0.021 - 0.074) | (-0.058 - 0.124) |
|  | p | 0.576 | 0.011 | 0.006 | 0.169 | 0.274 | 0.473 |
| Number of MRI targets | Coef. | 3.103 | 2.074 | 6.806 | 1.083 | 2.422 | 2.452 |
|  | 95%CI | (1.840 - 4.367) | (1.770 - 2.377) | (3.697 - 9.914) | (-1.301 - 3.466) | (1.812 - 3.032) | (1.656 - 3.248) |
|  | p | 0.000 | 0.000 | 0.000 | 0.369 | 0.000 | 0.000 |
| Pain NRS (0-10) | Coef. | 0.299 | -0.051 | 0.328 | 0.339 | -0.208 | -0.076 |
|  | 95%CI | (0.070 - 0.529) | (-0.156 - 0.054) | (-0.264 - 0.919) | (-0.049 - 0.726) | (-0.418 - 0.003) | (-0.408 - 0.257) |
|  | p | 0.011 | 0.339 | 0.272 | 0.086 | 0.053 | 0.652 |
| PIRADS 4 lesion § | Coef. | 0.797 | 0.404 | -2.290 | 1.660 | -0.197 | 0.244 |
|  | 95%CI | (-0.776 - 2.369) | (-0.026 - 0.834) | (-6.269 - 1.690) | (-0.947 - 4.267) | (-1.051 - 0.658) | (-1.086 - 1.574) |
|  | p | 0.320 | 0.065 | 0.253 | 0.209 | 0.649 | 0.716 |
| PIRADS 5 lesion § | Coef. | -0.079 | 0.668 | -5.240 | 1.471 | 0.900 | -0.375 |
|  | 95%CI | (-2.040 - 1.882) | (0.026 - 1.311) | (-9.906 - -0.574) | (-1.753 - 4.695) | (-0.309 - 2.109) | (-2.281 - 1.531) |
|  | p | 0.937 | 0.042 | 0.028 | 0.367 | 0.143 | 0.697 |
| Prostate volume [cc] | Coef. | 0.004 | -0.012 | -0.030 | -0.005 | -0.006 | -0.014 |
|  | 95%CI | (-0.018 - 0.026) | (-0.019 - -0.005) | (-0.087 - 0.026) | (-0.035 - 0.025) | (-0.017 - 0.004) | (-0.046 - 0.019) |
|  | p | 0.723 | 0.001 | 0.289 | 0.741 | 0.204 | 0.411 |

| B.  csCDR-T –  multivariable regression |  | Centre 1 | Centre 2 | Operator 1 | Operator 2 | Operator 3 | Operator 5* |
| --- | --- | --- | --- | --- | --- | --- | --- |
| Age | OR | 1.082 | 1.081 | 1.045 | 1.176 | 1.004 | 1.332 |
|  | 95%-CI | (1.042 - 1.123) | (1.038 - 1.126) | (0.925 - 1.180) | (1.058 - 1.307) | (0.874 - 1.154) | (1.011 - 1.755) |
|  | p | 0.000 | 0.000 | 0.480 | 0.003 | 0.950 | 0.042 |
| Centre experience | OR | 1.002 | 1.004 |  |  |  |  |
|  | 95%-CI | (1.000 - 1.004) | (1.002 - 1.006) |  |  |  |  |
|  | p | 0.088 | 0.000 |  |  |  |  |
| Operator experience | OR |  |  | 1.000 | 1.023 | 0.984 | 1.037 |
|  | 95%-CI |  |  | (0.963 - 1.038) | (0.998 - 1.050) | (0.947 - 1.024) | (0.994 - 1.081) |
|  | p |  |  | 0.984 | 0.075 | 0.427 | 0.094 |
| PSA density [ng/ml/cc] | OR | 222.952 | 43.973 | 0.722 | 3,120.352 | 46.474 | 2,840.006 |
|  | 95%-CI | (6.712 - 7,405.414) | (7.429 - 260.274) | (0.001 - 671.349) | (1.301 - 7486327.771) | (0.081 - 26,826.559) | (0.791 - 10196550.885) |
|  | p | 0.002 | 0.000 | 0.926 | 0.043 | 0.237 | 0.057 |
| Prostate volume [cc] | OR | 0.979 | 0.980 | 0.946 | 0.990 | 0.970 | 0.955 |
|  | 95%-CI | (0.967 - 0.992) | (0.964 - 0.997) | (0.903 - 0.991) | (0.967 - 1.014) | (0.902 - 1.042) | (0.881 - 1.036) |
|  | p | 0.001 | 0.024 | 0.019 | 0.407 | 0.402 | 0.266 |
| Positive DRE | OR | 2.376 | 2.954 | 5.297 | 5.643 | 1.961 | 26.384 |
|  | 95%-CI | (1.367 - 4.130) | (1.533 - 5.693) | (1.089 - 25.767) | (1.444 - 22.054) | (0.210 - 18.309) | (1.464 - 475.474) |
|  | p | 0.002 | 0.001 | 0.039 | 0.013 | 0.555 | 0.027 |
| PI-RADS 4 lesion § | OR | 3.861 | 5.224 | 1.730 | 5.383 | 3.709 | 3.997 |
|  | 95%-CI | (1.751 - 8.518) | (2.553 - 10.688) | (0.183 - 16.375) | (1.002 - 28.911) | (0.289 - 47.561) | (0.109 - 146.205) |
|  | p | 0.001 | 0.000 | 0.633 | 0.050 | 0.314 | 0.451 |
| PI-RADS 5 lesion § | OR | 3.314 | 8.444 | 71.576 | 3.931 | 2.084 | 237.558 |
|  | 95%-CI | (1.163 - 9.443) | (3.216 - 22.172) | (1.295 - 3,955.850) | (0.456 - 33.853) | (0.070 - 61.931) | (3.083 - 18,306.001) |
|  | p | 0.025 | 0.000 | 0.037 | 0.213 | 0.671 | 0.014 |
| Target diameter [mm] | OR | 1.157 | 1.009 | 0.837 | 1.245 | 1.087 | 0.887 |
|  | 95%-CI | (1.074 - 1.247) | (0.956 - 1.065) | (0.652 - 1.074) | (1.054 - 1.469) | (0.912 - 1.296) | (0.708 - 1.110) |
|  | p | 0.000 | 0.752 | 0.161 | 0.010 | 0.353 | 0.294 |
| Positive family history | OR | 0.812 | 3.822 | 0.211 | 0.825 | - | 61.396 |
|  | 95%-CI | (0.382 - 1.725) | (1.206 - 12.111) | (0.017 - 2.590) | (0.172 - 3.951) |  | (2.952 - 1,277.003) |
|  | p | 0.587 | 0.023 | 0.224 | 0.809 |  | 0.008 |

.
